# Appendiceal Mucinous Neoplasms and Pseudomyxoma Peritonei: Current Classification and the Role of Intraperitoneal Chemotherapy

**DOI:** 10.3390/cancers18121999

**Published:** 2026-06-19

**Authors:** Walter Giuseppe Giordano, Giovanbattista Musumeci, Enrica Nasso, Alessandra Briguglio, Ferdinando Macrì, Angela D’Ascola, Antonio Ieni, Antonio Macrì

**Affiliations:** 1 Department of Human Pathology of the Adulthood and Developing Age “Gaetano Barresi”, Section of Pathology, University of Messina, 98122 Messina, Italy; ferdinando.macri.2110@gmail.com (F.M.); antonio.ieni@unime.it (A.I.); 2 General and Emergency Surgery Unit, Azienda Ospedaliera Universitaria “G. Martino”—Messina, 98125 Messina, Italy; gbmusumeci@gmail.com (G.M.); briguglioalessandra@gmail.com (A.B.); antonio.macri@unime.it (A.M.); 3 Department of Clinical and Experimental Medicine, Policlinic “G. Martino”, University of Messina, 98122 Messina, Italy; enrica@nasso.it (E.N.); angela.dascola@unime.it (A.D.)

**Keywords:** pseudomyxoma peritonei, appendiceal mucinous neoplasm, appendiceal neoplasms, peritoneal surface malignancy, cytoreductive surgery, hyperthermic intraperitoneal chemotherapy, pressurized intraperitoneal aerosol chemotherapy, tumor organoids, precision oncology, *KRAS*

## Abstract

Appendiceal mucinous neoplasms (AMNs) are a rare but clinically significant category of gastrointestinal tumors, ranging from low-grade appendiceal mucinous neoplasm (LAMN), the main precursor of pseudomyxoma peritonei (PMP), to high-grade appendiceal mucinous neoplasm (HAMN), poorly differentiated and signet-ring-cell adenocarcinomas, and goblet cell adenocarcinoma. Although current WHO and PSOGI classifications provide well established diagnostic criteria, controversies persist regarding the biological behavior and prognostic significance of the most aggressive subtypes and the relationship between HAMN and mucinous adenocarcinoma. While appendectomy is sufficient for localized LAMN, cytoreductive surgery with hyperthermic intraperitoneal chemotherapy (CRS/HIPEC) is the treatment of choice for peritoneal dissemination This review integrates the histopathological and molecular classification of AMN and PMP with the evolution of intraperitoneal chemotherapy. Key findings indicate that KRAS and GNAS mutations are central drivers of mucin overproduction and peritoneal spread, that tumor grade and mucin cellularity remain the strongest prognostic determinants, and that the evidence supporting HIPEC and PIPAC derives largely from observational rather than randomized data. As a novel insight, we highlight the emerging role of patient-derived organoids as translational models for functional drug testing. Progress will depend on integrating molecular characterization, critical appraisal of intraperitoneal therapies, and organoid-based testing to advance individualized treatment for peritoneal surface malignancies.

## Simple Summary

Appendiceal mucinous neoplasms (AMNs) and pseudomyxoma peritonei (PMP) are rare malignancies characterized by mucin production and progressive dissemination within the peritoneal cavity. Over the past decades, the combination of cytoreductive surgery (CRS) and hyperthermic intraperitoneal chemotherapy (HIPEC) has significantly improved patient survival and represents the current standard of care for selected patients. However, recurrence remains common, particularly in high-grade disease, and the biological mechanisms underlying tumor progression and therapeutic resistance are still incompletely understood. Despite numerous reviews addressing the surgical management of these tumors, an integrated perspective that links their evolving histopathological and molecular classification to current and emerging intraperitoneal treatment strategies is still lacking. This review addresses that gap by bringing together, in a single framework, the current classification of AMNs and PMP, the role and evolution of intraperitoneal chemotherapy strategies including HIPEC and PIPAC, and the molecular alterations that drive disease behavior. Its main novelty lies in connecting these molecular insights with emerging translational approaches, particularly patient-derived organoids, which may improve preclinical modelling, drug testing, and personalized treatment selection. Together, these advances support the transition toward more biologically informed and individualized therapeutic strategies for peritoneal surface malignancies.

## 1. Introduction

Peritoneal surface malignancies comprise a heterogeneous group of tumors that either arise primarily from the peritoneum or disseminate to it from other organs. They are conventionally divided into primary peritoneal tumors, such as malignant peritoneal mesothelioma, and secondary peritoneal involvement, which most commonly originates from ovarian, gastric, colorectal, pancreatic, and appendiceal cancers [1]. Although historically associated with a uniformly poor prognosis, the introduction of cytoreductive surgery (CRS) combined with hyperthermic intraperitoneal chemotherapy (HIPEC) has substantially improved outcomes in selected patients, and this combined approach is now regarded as the standard of care for several of these conditions, particularly pseudomyxoma peritonei and peritoneal mesothelioma [1]. Within this broad spectrum, appendiceal mucinous neoplasms and their peritoneal counterpart, pseudomyxoma peritonei, represent a biologically distinct subgroup whose indolent yet relentless behavior poses unique diagnostic and therapeutic challenges.

Appendiceal mucinous neoplasms (AMN) are rare epithelial tumors, accounting for less than 1% of gastrointestinal malignancies. Although rare, the incidence of appendiceal cancers has risen substantially over the past two decades, increasing by approximately 54% between 2000 and 2009 in population-based data, with mucinous adenocarcinoma representing the most common histological subtype [2]. These tumors are frequently discovered incidentally, either during surgery for suspected appendicitis or on imaging performed for nonspecific abdominal complaints. When mucin-producing neoplastic epithelium disseminates within the peritoneal cavity, it gives rise to pseudomyxoma peritonei (PMP), a rare condition with an estimated incidence of approximately one to two cases per million per year, characterized by progressive accumulation of mucinous ascites and diffuse peritoneal implants [3]. Notably, PMP develops in approximately 20% of patients with mucinous appendiceal neoplasms, underscoring the clinical relevance of accurate early recognition [3].

The clinical management of AMN and PMP is complicated by several factors. Their rarity limits the availability of high-level evidence and concentrates expertise in a small number of specialized centers. The insidious clinical course often delays diagnosis until advanced peritoneal dissemination has occurred, and even after optimal treatment, recurrence remains common, particularly in high-grade disease. Moreover, the extensive surgical procedures required for cytoreduction carry significant morbidity, while the unique mucinous microenvironment hampers the penetration and efficacy of systemic and intraperitoneal chemotherapy. Since the introduction of CRS and HIPEC in the 1980s, the prognosis and quality of life of these patients have improved considerably; nevertheless, standardized treatment protocols for AMN, and especially for cases complicated by PMP, are still lacking [4,5,6].

In recent years, knowledge of the biological characteristics of these tumors has expanded considerably, prompting major revisions of diagnostic and prognostic criteria. The World Health Organization (WHO) and the Peritoneal Surface Oncology Group International (PSOGI) have developed shared guidelines that have substantially improved the standardization of diagnosis. Despite these advances, several controversies and knowledge gaps persist. The classification of the most aggressive and less common subtypes remains incompletely defined, the molecular mechanisms driving progression from localized neoplasm to peritoneal dissemination are only partially understood, and the relative roles of HIPEC and emerging strategies such as pressurized intraperitoneal aerosol chemotherapy (PIPAC) remain to be firmly established through high-level evidence. These unresolved issues highlight the need for an integrated perspective linking the evolving histopathological and molecular classification of these tumors to current and emerging therapeutic approaches.

Accordingly, the aim of this review is to provide a comprehensive and critical overview of appendiceal mucinous neoplasms and pseudomyxoma peritonei, bridging pathology, molecular biology, and clinical management. Specifically, this review addresses four key questions: (i) how the classification of AMN and PMP has evolved and which areas remain controversial; (ii) which molecular alterations drive their biological behavior and dissemination; (iii) what is the current evidence supporting intraperitoneal chemotherapy strategies, including HIPEC and PIPAC, and what are their limitations; and (iv) how emerging translational models, particularly patient-derived organoids, may improve prognostic stratification and enable individualized treatment. By integrating these perspectives, the review aims to contextualize current practice within a biologically informed framework and to identify directions for future research.

### Search Strategy

This narrative review is based on a comprehensive literature search of the PubMed/MEDLINE database, covering publications from 1980 to 2025. The search combined the following keywords and their variations: “appendiceal mucinous neoplasm”, “LAMN”, “HAMN”, “pseudomyxoma peritonei”, “appendiceal adenocarcinoma”, “peritoneal surface malignancy”, “cytoreductive surgery”, “HIPEC”, “PIPAC”, “molecular pathogenesis”, “KRAS”, “GNAS”, and “tumor organoids”. Both original research articles and reviews published in English were considered, with priority given to recent, high-quality, and clinically relevant publications, including international consensus statements and guidelines. Seminal earlier works were also retained for their historical and conceptual relevance. The reference lists of selected articles were manually screened to identify additional pertinent studies.

## 2. Classification and Major Histologic Types

### 2.1. Evolution of Terminology and Classification Frameworks

The classification of appendiceal mucinous neoplasms (AMNs) has undergone profound changes over the past several decades, reflecting the gradual understanding of their biological behavior and relationship with peritoneal disease. Historically, a variety of confusing and often overlapping terms such as mucocele, mucinous cystadenoma, mucinous cystadenocarcinoma, and mucosal hyperplasia were used to describe a wide spectrum of mucin-producing lesions of the appendix. These designations, based primarily on descriptive morphology rather than biological potential, led to significant inconsistencies in diagnosis, reporting, and clinical management. An overview of the historical terms and their correspondence to the current nomenclature is provided in Table 1.

In earlier decades, all cystic dilatations of the appendix filled with mucin were generically referred to as mucoceles. However, this term failed to distinguish between simple non-neoplastic processes and true neoplasms capable of peritoneal dissemination. As more cases were studied and clinical follow-up data accumulated, it became increasingly clear that certain appendiceal mucinous lesions behaved in an indolent manner, while others led to diffuse peritoneal spread in the form of pseudomyxoma peritonei (PMP). This observation underscored the need for a more precise histopathologic classification system capable of correlating morphology with prognosis.

A major step toward standardization came with the World Health Organization (WHO) Classification of Digestive System Tumors, 5th Edition (2019). This edition formally introduced two distinct categories Low-Grade Appendiceal Mucinous Neoplasm (LAMN) and High-Grade Appendiceal Mucinous Neoplasm (HAMN) which replaced the older terminology of mucinous cystadenoma and cystadenocarcinoma. These categories emphasize the cytologic grade and pattern of invasion, two features that most accurately predict the biological behavior of the tumors. According to the WHO, LAMNs and HAMNs are classified as mucinous epithelial tumors of uncertain or borderline malignant potential, assigned with specific ICD-O3 behavior codes: 8480/1 for LAMN (low malignant potential) and 8480/2 for HAMN (high-grade, borderline malignant) [7,8,9,10]. This classification as tumors of uncertain or borderline malignant potential reflects a fundamental feature of these neoplasms: despite their bland or only moderately atypical histology, they retain the capacity to disseminate within the peritoneal cavity. Importantly, LAMN and HAMN differ substantially in their biological behavior. LAMNs generally follow an indolent course and, when confined to the appendix, carry a low risk of peritoneal spread; their principal mechanism of dissemination is rupture or compromise of the appendiceal wall, which allows mucin and neoplastic epithelium to seed the peritoneum and give rise to pseudomyxoma peritonei (PMP) [10,11]. In contrast, HAMNs exhibit high-grade cytology, elevated proliferative activity, and abnormal p53 expression, corresponding to a more aggressive phenotype with a greater propensity for peritoneal dissemination even in the absence of overt invasion [12,13]. This distinction is clinically relevant, as tumor grade not only influences the likelihood of progression to PMP but also determines prognosis once peritoneal disease is established, with low-grade disease carrying a substantially more favorable outcome than its high-grade counterpart [8]. The defining characteristic of both entities, therefore, is not invasion in the conventional sense but their capacity for peritoneal dissemination, which underlies their classification as lesions of borderline malignant potential.

In parallel, the Peritoneal Surface Oncology Group International (PSOGI) published a consensus statement in 2016, which further refined the histopathologic definitions and linked appendiceal neoplasms to the spectrum of peritoneal mucinous disease. The PSOGI framework unified terminology across institutions and introduced the concept of a continuum between appendiceal mucinous tumors and pseudomyxoma peritonei. This classification emphasized not only the epithelial grade but also the cellularity of the mucin within the peritoneal cavity, a key determinant of prognosis [8].

The American Joint Committee on Cancer (AJCC) 8th Edition also contributed to this process by including, for the first time, specific staging criteria for appendiceal mucinous neoplasms. These criteria account for the extent of mural invasion, the presence of acellular or cellular mucin outside the appendix, and peritoneal involvement [14]. This staging system has been instrumental in aligning pathologic assessment with clinical decision-making, particularly regarding the indication for cytoreductive surgery and hyperthermic intraperitoneal chemotherapy (HIPEC).

Together, these coordinated efforts by the WHO, PSOGI, and AJCC have led to a modern, biologically meaningful classification of appendiceal mucinous neoplasms. This unified framework allows for consistent diagnosis, facilitates accurate prognostication, and supports more rational therapeutic planning. The evolution of terminology from vague descriptive labels to a refined histopathologic and molecularly informed system represents one of the most significant progresses in the management of these rare and complex tumors.

### 2.2. Serrated Lesions

Among the spectrum of epithelial abnormalities in the appendix, serrated lesions occupy a unique and somewhat enigmatic niche. Their recognition, classification, and potential role as precursor lesions to mucinous neoplasms have drawn increasing attention in recent years. While historically they were underappreciated (often regarded as incidental or of minimal clinical significance), emerging pathological and molecular evidence suggests that the appendiceal serrated pathway deserves careful consideration.

#### 2.2.1. Definition and Subclassifications

Serrated lesions of the appendix represent a heterogeneous group of mucinous epithelial proliferations characterized by a distinctive “saw-tooth” or serrated crypt architecture, closely resembling their colonic counterparts but differing significantly in biological behavior, molecular alterations, and clinical implications.

According to the 2019 World Health Organization (WHO) classification of digestive system tumors, serrated lesions of the appendix are subdivided into three principal histopathologic categories based on the degree of serration and presence or absence of cytologic dysplasia [7,8]. Hyperplastic polyp (HP) are benign lesions demonstrating luminal serration limited to the superficial portion of the crypts, without cytologic atypia or dysplasia. They often arise incidentally and are not associated with significant malignant potential.

Serrated lesion without dysplasia (SL) exhibits more extensive serration involving the entire length of the crypt, yet retains cytologic blandness, without evidence of dysplasia. Although histologically like sessile serrated lesions (SSLs) in the colon, their molecular profile is distinct, and they are rarely linked to the serrated neoplasia pathway commonly described in colorectal carcinogenesis [7]. Serrated lesion with dysplasia (SLD) present a serrated architecture in conjunction with focal or multifocal cytologic dysplasia, which may assume adenoma-like, serrated, or mixed morphologic patterns. The presence of dysplasia signifies neoplastic transformation and may represent a precursor stage to LAMNs in rare cases [9].

From a pathobiological perspective, while the classification system mirrors that of colonic serrated polyps, appendiceal serrated lesions are notably less frequent and demonstrate distinct molecular signatures. For example, *BRAF* mutations commonly associated with colonic serrated lesions are infrequent in the appendix, whereas *KRAS* mutations predominate, suggesting divergent oncogenic pathways [7,8,9]. Furthermore, the anatomic environment of the appendix, with its narrow lumen and high secretory activity, may favor mucin retention and cystic dilatation, occasionally mimicking or coexisting with mucinous neoplasms.

Clinically, these lesions are usually asymptomatic and discovered incidentally during appendectomy for unrelated conditions. However, in some cases, particularly SLDs, localized mural expansion or mucin accumulation can occur, raising suspicion for an early-stage mucinous neoplasm. Careful histopathologic distinction between SLD and LAMN is therefore essential, as the latter implies a neoplastic process with potential for dissemination if ruptured [7].

In summary, serrated appendiceal lesions represent an uncommon but distinct subset of mucinous proliferations whose recognition is essential for accurate classification, prognostication, and prevention of overtreatment. Understanding their subtle architectural and molecular differences from colonic serrated polyps enhances diagnostic precision and underscores the appendix’s unique tumor biology [10,14].

#### 2.2.2. Morphological Characteristics and Histological Evidence

In HPs, the serration is relatively mild and limited to the luminal surfaces, and the deeper crypt structure, lamina propria, and muscularis mucosae remain intact. In SLs, the crypts often show distortion, dilation, and extension of serration toward the crypt base, sometimes with L-shaped or inverted T-shaped formations. Yet even in SLs, the mucosa retains its full complement of lamina propria and muscularis mucosae, and no stromal invasion or mucin penetration into the wall is present [8].

When dysplasia is present (SLD), one may observe cytologic changes such as nuclear enlargement, hyperchromasia, loss of polarity, and mitoses. These dysplastic areas may be patchy, and multiple dysplasia morphologies (serrated-type, adenomatous-type) can coexist in a single lesion [9]. Importantly, despite these focal high-risk features, the lesion still lacks overt invasive behavior.

One challenge is that some areas of LAMNs may display focal serrated architecture, blurring the boundary between serrated lesions and mucinous neoplasia. Distinguishing these reliably requires evaluation of additional features presence or absence of lamina propria, integrity of crypt architecture, mural mucin/fibrosis, and whether mucin dissects into the appendiceal wall [9,10].

#### 2.2.3. Incidence and Detection

Serrated lesions in the appendix tend to be incidental frequently discovered during pathologic examination after appendectomy for presumed appendicitis. Because these lesions are often small and show no gross change in the appendix, they are easily missed in routine partial sampling [9]. Indeed, studies have shown that when the entire appendix is submitted for histology, the detection rate of serrated lesions rises significantly compared to partial sampling protocols [9].

This underdiagnosis is clinically relevant not only because of the need to distinguish benign serrated lesions from mucinous neoplasia, but also because of the potential overlap in molecular features and morphological mimicry between serrated lesions and early mucinous tumors [8,15].

#### 2.2.4. Molecular Correlations in Serrated Lesions

Molecular studies suggest that the *KRAS* mutation is common to both serrated lesions and mucinous neoplasms of the appendix, hinting at a possible shared neoplastic pathway. However, the classic *BRAF* mutations seen in colorectal serrated carcinogenesis are far less common in appendiceal lesions, indicating a divergent serrated biology in the appendix [8,11,15].

A recent study by Mahadik et al. [15] found that among 27 LAMNs, 8 (29.6%) exhibited serrated architecture (HP-like, SSL-like, or TSA-like), and among serrated polyps, a few cases overlapped morphologically with mucinous neoplasms. All such overlapping cases harbored *KRAS* mutations [15]. This supports the notion that serrated polyps and LAMNs may exist along a biological continuum. Despite growing interest, the role of serrated lesions in appendiceal tumorigenesis remains controversial and incompletely understood. Unlike the well-characterized serrated neoplasia pathway of the colon, in which *BRAF* mutation and the CpG island methylator phenotype drive progression toward microsatellite-unstable carcinoma, appendiceal serrated lesions are predominantly *KRAS*-mutated and rarely harbor *BRAF* alterations, indicating a fundamentally distinct biology whose significance is not yet resolved [16]. A central unresolved question is whether appendiceal serrated lesions represent genuine precursors of mucinous neoplasms or merely morphological mimics that share a common KRAS-driven background. The frequent co-occurrence of serrated lesions and LAMN, together with the overlapping *KRAS* mutational profile, has led some authors to propose a biological continuum in which a subset of LAMNs may arise from precursor serrated lesions, possibly through additional *GNAS* or WNT-pathway alterations [15,16]. However, this hypothesis remains unproven: GNAS mutations, characteristic of LAMN, have not been systematically demonstrated in appendiceal serrated lesions, and direct longitudinal evidence of progression is lacking. The rarity of these lesions, the absence of large molecularly annotated cohorts, and the considerable morphological overlap that complicates reproducible classification all contribute to the persistent uncertainty. Until larger, molecularly characterized series become available, the precise position of serrated lesions within the spectrum of appendiceal tumorigenesis will remain an open question.

#### 2.2.5. Clinical Implications, Differential Diagnosis, and Management

**Figure 1:**
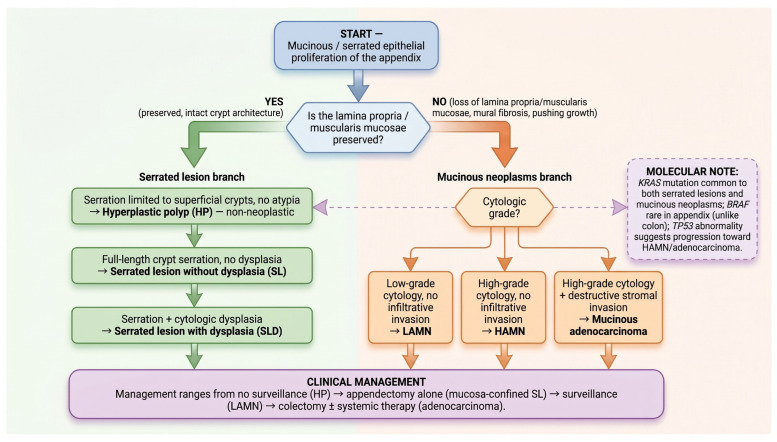
Diagnostic algorithm for the differential diagnosis of appendiceal mucinous and serrated epithelial lesions. The integrity of the lamina propria and muscularis mucosae represents the key discriminating feature: preserved crypt architecture characterizes serrated lesions (hyperplastic polyp, serrated lesion without dysplasia, and serrated lesion with dysplasia), whereas its loss, together with mural fibrosis and a pushing growth pattern, defines mucinous neoplasms, further stratified by cytologic grade and the presence of infiltrative invasion into LAMN, HAMN, and mucinous adenocarcinoma. The molecular note summarizes the shared and distinguishing genetic alterations, and the lower panel outlines the corresponding clinical management. HP, hyperplastic polyp; SL, serrated lesion without dysplasia; SLD, serrated lesion with dysplasia; LAMN, low-grade appendiceal mucinous neoplasm; HAMN, high-grade appendiceal mucinous neoplasm. This figure is an original creation by the authors and has not been reproduced or adapted from previously published sources. The dashed arrow indicates a proposed biological continuum between serrated lesions and mucinous neoplasms, based on shared KRAS mutational features.

Discriminating true serrated lesions from early LAMN is not merely semantic it has implications for prognosis, follow-up, and therapeutic choice. Because LAMNs carry a risk of peritoneal dissemination, overcalling a serrated lesion as LAMN may lead to overtreatment; conversely, underrecognizing LAMN may delay surveillance or intervention. Several key features favor LAMN over serrated lesions, including the loss or obliteration of the lamina propria and muscularis mucosae, mucin dissection into the wall or breach of the serosa, submucosal fibrosis or stromal reaction, and flattened or villous epithelial growth replacing the native mucosa rather than merely serration [9,10] [Figure 1].

From a clinical standpoint, the management and surveillance of appendiceal serrated lesions are dictated primarily by the presence and grade of dysplasia and by the integrity of the appendiceal wall. Non-neoplastic changes such as diffuse mucosal hyperplasia, which often arise in the setting of resolving appendicitis, require no further treatment or surveillance, whereas serrated neoplasms confined to the mucosa are generally considered adequately treated by appendectomy alone, if resection is complete [17]. Lesions exhibiting dysplasia (SLD), or those that cannot be confidently distinguished from early LAMN, warrant closer attention, since correct categorization directly influences the need for surveillance and the risk of subsequent peritoneal disease [17]. A further clinically relevant consideration is the documented association between appendiceal serrated polyps and synchronous or metachronous colorectal neoplasia: the yield of advanced colorectal lesions in these patients has been reported to be several-fold higher than in the general screening population, leading some authors to recommend complete colonoscopy following the incidental discovery of an appendiceal serrated lesion [18]. Finally, because these lesions are frequently small and easily overlooked, complete submission and thorough histologic examination of the appendectomy specimen are essential to avoid underdiagnosis and to guide appropriate follow-up [9]. Taken together, these considerations underscore that, although most appendiceal serrated lesions are indolent, their accurate recognition carries tangible implications for patient management, ranging from no intervention to colonoscopic screening and, in selected cases, structured surveillance.

### 2.3. Mucinous Neoplasms of the Appendix (LAMN and HAMN)

Mucinous neoplasms of the appendix represent a distinctive subset of epithelial appendiceal tumors characterized by abundant extracellular mucin production and a gland-forming mucinous epithelium that progressively replaces the normal appendiceal mucosa. These tumors have garnered increasing attention over the past two decades due to their unique morphology, unpredictable clinical course, and their strong association with Pseudomyxoma Peritonei (PMP) a rare but serious peritoneal dissemination syndrome [8]. Historically, appendiceal mucinous tumors were classified using terms such as mucinous cystadenoma, mucinous cystadenocarcinoma, or mucocele. However, these designations failed to accurately capture the biological continuum between benign, borderline, and malignant forms, often leading to diagnostic inconsistencies and misinterpretation of prognosis [8,9]. The evolution of histopathologic criteria, coupled with advances in molecular pathology, has clarified that these tumors do not always fit neatly into the traditional benign–malignant dichotomy [10,12].

**Figure 2:**
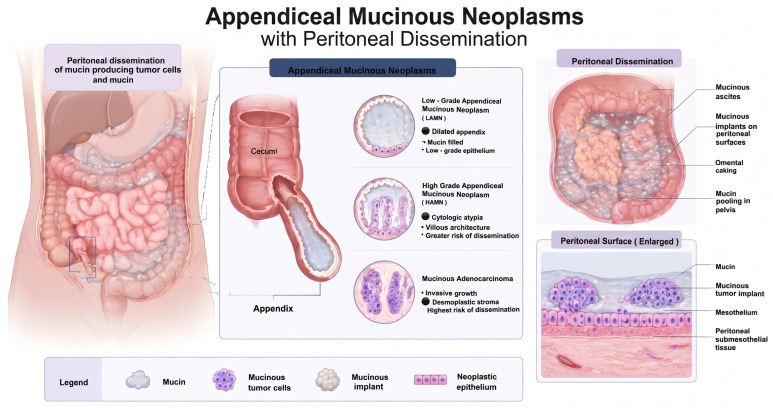
Pathogenesis and peritoneal dissemination of appendiceal mucinous neoplasms. Illustrative representation of the biological progression and peritoneal dissemination of AMNs, encompassing LAMN, HAMN, and mucinous adenocarcinoma. The figure elucidates mucin production and appendiceal dilation, subsequently followed by the dissemination of mucinous tumor cells into the peritoneal cavity, resulting in mucin accumulation, peritoneal implants, and pseudomyxoma peritonei-like disease. Histologic and schematic representations underscore the correlation between tumor grade, invasive potential, and the risk of peritoneal dissemination. This figure is an original creation by the authors and has not been reproduced or adapted from previously published sources.

According to the World Health Organization (WHO, 5th edition, 2019) and the Peritoneal Surface Oncology Group International (PSOGI) classification system, mucinous neoplasms of the appendix are now categorized into LAMN, HAMN, Mucinous Adenocarcinoma and Signet Ring Cell Carcinoma and Poorly Differentiated Variants [7,19]. These entities are differentiated based on cytologic grade and pattern of invasion rather than merely on mucin production or architectural features. Importantly, both LAMN and HAMN are considered non-infiltrative mucinous neoplasms, as they typically lack the destructive stromal invasion seen in mucinous adenocarcinomas, yet they may still exhibit peritoneal dissemination and recurrence, particularly when rupture of the appendiceal wall occurs [19,20] [Figure 2].

This apparent paradox, namely neoplasms capable of peritoneal spread despite the absence of true infiltrative invasion, represents one of the most distinctive biological features of these tumors and sets them apart from conventional adenocarcinomas. Rather than infiltrating the wall through a destructive, stroma-invading front, LAMN and HAMN characteristically extend by a “pushing” pattern of invasion: a broad, expansile advancing margin that displaces rather than destroys the surrounding tissue, and that may dissect acellular mucin or neoplastic epithelium into or through the appendiceal wall, occasionally mimicking a diverticulum [19,20]. Crucially, dissemination does not occur through the hematogenous or lymphatic routes typical of invasive carcinomas; instead, it follows a transcoelomic mechanism, whereby breach or rupture of the appendiceal wall releases mucin and viable neoplastic cells directly into the peritoneal cavity, where they implant and continue to secrete mucin, giving rise to pseudomyxoma peritonei [19,20]. The biological consequence of this mode of spread is that the nature of the extra-appendiceal material, rather than conventional invasion, governs prognosis: dissemination limited to acellular mucin carries a low risk of progression, whereas the presence of neoplastic epithelial cells within the peritoneal mucin confers a substantially higher risk of recurrence and established PMP [8]. This explains why staging and risk stratification of these neoplasms rely on the integrity of the appendiceal wall and the cellularity of any extra-appendiceal mucin, rather than on depth of infiltrative invasion as in most gastrointestinal malignancies.

### 2.4. Low-Grade Appendiceal Mucinous Neoplasm (LAMN)

#### 2.4.1. Definition and General Characteristics

LAMNs represents a distinct clinicopathologic entity within the spectrum of appendiceal mucinous tumors, defined by low-grade cytologic atypia, abundant extracellular mucin, and a characteristic “pushing” growth pattern that replaces rather than infiltrates the appendiceal wall [9,10]. These neoplasms arise from the appendiceal epithelium and are typified by mucinous proliferation that leads to progressive dilatation of the lumen and replacement of the normal mucosal architecture by fibrotic, hyalinized stroma [8].

LAMN occupies an intermediate biological position between simple mucosal hyperplasia and invasive mucinous adenocarcinoma. While it lacks destructive invasion, it has the potential for peritoneal dissemination through mucin extravasation when the appendiceal wall becomes compromised [10].

The clinical relevance of LAMN has increased significantly in the last two decades due to its strong association with pseudomyxoma peritonei (PMP), a condition characterized by the accumulation of mucin within the peritoneal cavity. Consequently, correct histologic classification of LAMN is critical for determining prognosis and guiding surgical management.

#### 2.4.2. Histopathologic Features

Macroscopically, LAMNs typically present as distended, mucus-filled appendices often with thin, fibrotic, or translucent walls [10]. The luminal diameter frequently exceeds 2–3 cm and the cavity is filled with viscous, gelatinous mucin [9]. In advanced cases, mural thinning and focal rupture may be observed, occasionally leading to mucin spillage into the peritoneal cavity [11].

Microscopically, the hallmark features of LAMN include a villiform or undulating mucinous epithelium composed of tall, columnar goblet-like cells with basally located nuclei and abundant apical mucin [9]. There is a loss of the lamina propria and muscularis mucosae, which are replaced by dense hyalinized or fibrotic stroma [11]. The growth pattern is pushing and non-destructive, contrasting with the infiltrative glands of adenocarcinoma [8]. A common finding is the dissection of acellular mucin into or through the appendiceal wall, which reflects gradual mural weakening rather than invasion [12]. Sometimes a mural rupture with extravasation of mucin and, rarely, neoplastic epithelial cells in peri-appendicular fat may occur. Cytologic atypia is minimal, with elongated, uniform nuclei, fine chromatin, and inconspicuous nucleoli [10,12]. Mitotic figures are rare. These cytologic and architectural features are essential to distinguish LAMN from high-grade or invasive lesions, particularly in cases of peritoneal dissemination where tissue fragments may be scant.

#### 2.4.3. Immunohistochemistry

LAMNs display an immunophenotype consistent with intestinal and goblet cell differentiation. They characteristically express CK20, CDX2 and MUC2, confirming their intestinal-type mucinous nature [9,13]. MUC5AC, a gastric-type mucin, is also frequently expressed, reflecting the mixed intestinal–gastric phenotype that is characteristic of these neoplasms [21]. Expression of CK7 is usually negative or, at most, focal helping to differentiate LAMN from other lower gastrointestinal or ovarian mucinous neoplasms [9].

Proliferation and tumors suppressor markers further corroborate the indolent biology of these tumors. Ki-67 and p53 indices are typically low, with diffuse wild-type p53 staining and limited proliferative activity confined to the basal crypts [12]. This contrasts sharply with HAMN and adenocarcinomas, which show elevated proliferation indices and abnormal p53 accumulation. The immune profile thus serves both diagnostic and differential purposes particularly when distinguishing primary appendiceal lesions from secondary mucinous metastases to the peritoneum.

#### 2.4.4. Molecular Pathogenesis

At the molecular level, LAMNs are defined by co-activating mutations in *KRAS* (>90%) and *GNAS* (~70%), driving epithelial proliferation and mucin hypersecretion through the RAS–MAPK and cAMP–PKA pathways respectively [22,23]. *TP53* mutations are typically absent, consistent with the indolent biology of these neoplasms [13]. This combined *KRAS*/*GNAS* mutation signature is highly specific for appendiceal mucinous origin and serves as a powerful diagnostic tool to distinguish peritoneal lesions from metastatic colorectal adenocarcinoma, which rarely harbours *GNAS* mutations [9].

Beyond their diagnostic value, these molecular findings carry emerging prognostic and therapeutic implications. From a prognostic standpoint, mutation in *KRAS*, *GNAS*, or both has been associated with poorer survival in patients with peritoneal disease, although outcome remains more closely linked to the histological grade and extent of peritoneal involvement than to mutational status alone [24]. From a therapeutic perspective, the implications are twofold. First, the molecular distinction from colorectal cancer underscores that treatment regimens extrapolated from colorectal protocols may be poorly suited to these tumors, since their *KRAS*/*GNAS*-driven, mucin-rich biology differs fundamentally, which partly explains the limited efficacy of conventional systemic chemotherapy in this setting. Second, although *KRAS* and *GNAS* have historically been considered “undruggable”, the recent development of direct KRAS inhibitors and the growing interest in targeting *GNAS*-driven signaling have opened the possibility of molecularly rational therapies, which remain investigational but represent a promising direction for future studies. Together, these considerations reinforce the rationale for incorporating molecular profiling into the diagnostic and prognostic assessment of appendiceal mucinous neoplasms.

#### 2.4.5. Pathophysiology and Clinical Course

LAMNs are most often discovered incidentally during appendectomy for presumed acute appendicitis or other abdominal surgery [11]. Clinical manifestations, when present, include chronic right lower quadrant discomfort or a palpable mass. The indolent course reflects the slow accumulation of mucin and the absence of invasive growth.

When the tumors remain confined to the appendix, simple appendectomy is typically curative, with nearly 100% disease-specific survival [20,25]. However, rupture of the appendiceal wall can result in mucin extravasation into the peritoneal cavity a key determinant of prognosis [26,27].

Two distinct outcomes are recognized following rupture:

Acellular mucin spillage, which carries an excellent prognosis, as no viable epithelium is present to sustain peritoneal disease. Cellular mucin, containing viable neoplastic epithelial cells, which predisposes to PMP, a condition characterized by recurrent mucinous ascites and progressive peritoneal fibrosis [26,27].

These observations emphasize the critical importance of surgical handling. Intraoperative rupture should be strictly avoided, and cases with perforation require close radiologic and clinical monitoring.

#### 2.4.6. Prognosis and Management

The prognosis for LAMN confined to the appendix is exceptionally favorable, with near-complete long-term survival and minimal risk of recurrence [27]. Once extra-appendiceal mucin containing epithelial cells is identified, the clinical course becomes more variable, necessitating multidisciplinary management.

Current consensus recommends complete surgical excision without rupture and careful histologic examination of resection margins. For cases with peritoneal spread or PMP, cytoreductive surgery (CRS) combined with hyperthermic intraperitoneal chemotherapy (HIPEC) is the preferred therapeutic approach, as it offers significant improvement in survival and disease control [26,27].

Current guideline recommendations, including the PSOGI consensus, stratify management according to tumor stage and the presence and cellularity of extra-appendiceal mucin [28]. For LAMN confined to the appendix (pTis) with complete resection, appendectomy alone is considered curative, and no further surgery or systemic therapy is required. For tumors involving the serosa (pT4) but without extra-appendiceal mucin beyond the right lower quadrant and with complete (R0) resection, extended oncologic resection is not indicated and patients should enter structured surveillance; importantly, because LAMN does not metastasize through lymphatic or hematogenous routes, right hemicolectomy with lymphadenectomy is not justified [28]. When acellular or cellular mucin is identified on the appendiceal surface or within the right lower quadrant, the management of limited peritoneal involvement remains controversial and individualized, and CRS with or without HIPEC may be considered following multidisciplinary discussion, particularly when neoplastic epithelial cells are present [28]. In all cases, complete submission of the appendix for histopathologic examination is essential for accurate staging and risk stratification. For established peritoneal dissemination or PMP, CRS combined with HIPEC remains the treatment of choice. Given the absence of randomized data, these recommendations rest largely on expert consensus and observational evidence, and treatment decisions should be individualized within specialized multidisciplinary teams.

Long-term follow-up with periodic cross-sectional imaging is advised to detect early recurrence, especially in patients with prior rupture or residual mucin deposits.

LAMN thus represents a unique neoplastic model where biologic behavior is dictated not by invasion but by the integrity of the appendiceal wall and the cellularity of spilled mucin. Its recognition, classification, and management require close collaboration between pathologists, surgeons, and oncologists to optimize outcomes.

### 2.5. High-Grade Appendiceal Mucinous Neoplasm (HAMN)

#### 2.5.1. Definition and General Characteristics

HAMNs are characterized by high-grade cytologic atypia occurring in a non-invasive, LAMNs like architectural framework [9,10,12]. HAMNs represent the high-grade cytologic counterpart of LAMNs while retaining a non-invasive growth pattern. They exhibit epithelial features of high-grade dysplasia, including nuclear atypia, pleomorphism, loss of polarity, and increased mitotic activity [9,13]. Despite this, the growth remains expansile and lacks the infiltrative, desmoplastic stromal invasion typical of conventional adenocarcinoma [8,13]. The 2019 WHO Classification of Digestive System Tumors and the PSOGI both formally recognize HAMN as a distinct intermediate category, positioned biologically and morphologically between LAMN and invasive mucinous adenocarcinoma [8,13]. This classification acknowledges that appendiceal mucinous neoplasia represents a morphologic and molecular continuum rather than a binary benign–malignant distinction, and that HAMN plays a pivotal role in this neoplastic evolution.

#### 2.5.2. Histopathologic Features

Grossly, HAMNs often resemble their low-grade counterparts: a dilated appendix containing abundant viscous mucin, frequently with mural fibrosis or focal wall rupture [13]. However, the epithelial lining reveals a marked increase in cytologic atypia while preserving the architectural features of LAMN [13,22].

Microscopically, the epithelial architecture remains villiform, undulating, or flattened, often associated with mural hyalinization and fibrosis. The cytologic features, however, define the lesion: enlarged vesicular nuclei with coarse chromatin, prominent nucleoli, stratified nuclei extending to the luminal surface, and frequent mitotic figures [9,13].

Loss of nuclear polarity is a consistent finding, and occasional apoptotic bodies or luminal necrosis may be seen, but true infiltrative invasion is absent [9,11,13]. Mucin dissecting through the appendiceal wall remains largely acellular, a feature that distinguishes HAMN from invasive mucinous adenocarcinoma [29]. The transition between LAMN and HAMN may occur within the same lesion, with areas of low-grade cytology abruptly giving way to high-grade dysplastic foci [29]. For this reason, extensive sampling of the appendix is strongly recommended to accurately evaluate the highest-grade present and to rule out an associated invasive component [29].

#### 2.5.3. Immunohistochemistry

Immunohistochemically, HAMNs retain the intestinal-type profile characteristic of appendiceal mucinous neoplasms [13,22,26]. They consistently express CK20, CDX2, MUC2, and MUC5AC, confirming their intestinal and goblet cell differentiation [13,22,26].

However, compared with LAMNs, HAMNs typically exhibit higher proliferative activity, with Ki-67 indices ranging from 20% to 40%, and increased p53 expression, often with an aberrant “mutant” staining pattern [13,22,26]. These immunohistochemical changes parallel the histologic impression of higher-grade atypia and are useful adjuncts in distinguishing HAMN from LAMN in borderline cases [13,26]. Importantly, the retention of CK20/CDX2 expression and lack of diffuse CK7 reactivity also serve as molecular fingerprints confirming appendiceal origin, particularly in cases of peritoneal dissemination where primary site identification may be challenging [9,26].

#### 2.5.4. Molecular Pathogenesis

HAMNs retain the hallmark *KRAS* and *GNAS* mutations of LAMNs but additionally acquire *TP53* and *SMAD4* alterations and occasional WNT-pathway mutations, reflecting molecular progression toward high-grade cytology [22,23,25,27]. These secondary events confer genomic instability, positioning HAMN as a molecular intermediate between non-invasive and invasive mucinous neoplasia.

#### 2.5.5. Pathophysiology and Clinical Course

Although HAMNs remain non-invasive, they exhibit a higher risk of peritoneal spread and recurrence compared to LAMNs [20,27]. Clinically, HAMNs may present with right lower quadrant pain, chronic appendicitis-like symptoms, or as an incidental imaging finding characterized by appendiceal dilatation secondary to mucin accumulation. In a subset of cases, rupture or mucin leakage at diagnosis leads to pseudomyxoma peritonei (PMP), a complication associated with significant morbidity [26].

Despite their high-grade cytology, HAMNs often behave in an indolent fashion when confined to the appendix and completely resected. However, the presence of peritoneal mucin containing viable epithelial cells dramatically worsens prognosis and requires aggressive management [20]. The therapeutic cornerstone remains complete surgical resection without rupture. For disseminated disease, cytoreductive surgery (CRS) combined with hyperthermic intraperitoneal chemotherapy (HIPEC) is the standard of care, providing meaningful survival benefit in experienced centers [20].

Reported 5-year survival rates for HAMN vary between 70% and 90%, depending primarily on the extent of peritoneal involvement and the completeness of cytoreduction [25]. Given its potential for progression, close postoperative surveillance is essential, with regular cross-sectional imaging to detect early peritoneal recurrence.

#### 2.5.6. Prognosis and Management

The principal diagnostic challenge in HAMN lies in distinguishing it from well-differentiated mucinous adenocarcinoma, particularly when peritoneal spread or wall perforation is present [9,13,27]. While both may share cytologic high-grade features, the absence of destructive stromal invasion remains the defining criterion for HAMN. Careful evaluation of the interface between epithelium and stroma is therefore crucial. Immunohistochemical and molecular correlation can assist in borderline cases, especially when limited tissue is available or invasive foci are equivocal. Pathologists should be cautious not to overdiagnoses adenocarcinoma in the absence of clear invasion, as this may lead to unnecessary overtreatment. Conversely, under-recognition of high-grade cytology may underestimate the risk of recurrence or peritoneal dissemination.

Several interpretive challenges further complicate the diagnosis of HAMN and may contribute to variability between pathologists. Because the distinction from LAMN rests on the presence of high-grade cytologic atypia, which current criteria allow to be recognized even when focal provided it is unequivocal, the assessment retains a degree of subjectivity, and areas of low- and high-grade cytology may coexist within the same lesion [29]. This heterogeneity, together with the difficulty of evaluating fragmented or limited samples and of interpreting mucin or epithelium on the serosal surface in perforated specimens, can lead to inconsistent classification and to both over- and underdiagnosis [9,13]. For these reasons, extensive sampling of the appendix to identify the highest-grade focus, the use of standardized terminology and reporting criteria, and expert pathologic review in borderline cases are strongly recommended to improve diagnostic consistency [29].

### 2.6. Mucinous Adenocarcinoma of the Appendix

#### 2.6.1. Definition and General Characteristics

Mucinous adenocarcinoma of the appendix represents the invasive and malignant terminus of the appendiceal mucinous neoplasm spectrum. It is defined by the presence of destructive stromal invasion, complex glandular or cribriform architecture, and cytologic high-grade atypia [29,30].

In contrast to LAMN and HAMN, which are non-invasive, mucinous adenocarcinomas demonstrate unequivocal tissue infiltration and stromal desmoplasia, indicating a transition to frank malignancy. These tumors, though relatively rare, are clinically significant because they account for the majority of appendiceal carcinomas associated with PMP and have a markedly worse prognosis than their non-invasive counterparts [29,30]. Accurate histopathologic recognition and staging are therefore essential, as the therapeutic strategies and expected clinical outcomes differ profoundly between non-invasive mucinous neoplasms and invasive adenocarcinomas.

#### 2.6.2. Histopathologic Features

Histologically, mucinous adenocarcinoma of the appendix is characterized by irregular, infiltrative glands and cell clusters that permeate the appendiceal wall, frequently accompanied by a desmoplastic stromal reaction [31]. This invasive growth pattern distinguishes it sharply from the pushing, non-destructive architecture of LAMN and HAMN.

The neoplastic glands are typically angulated, cribriform, or micropapillary, and may form solid clusters or single cells floating within pools of extracellular mucin [18]. In many cases, more than 50% of the tumors volume consists of acellular mucin, a hallmark of the mucinous phenotype [31,32]. Areas with signet-ring cell morphology, defined by intracytoplasmic mucin displacing the nucleus peripherally, signify high-grade transformation and portend a more aggressive biological course [31].

The epithelial cells show moderate-to-marked nuclear pleomorphism, prominent nucleoli, and brisk mitotic activity [9,26]. Architectural heterogeneity is common: glandular, papillary, and micropapillary configurations often coexist within the same lesion [30,32].

Lympho-vascular and perineural invasion are frequent findings and correlate with advanced stage and poor outcome. Necrotic debris, luminal sloughing, and mucin lakes infiltrated by malignant epithelium are additional features supporting invasive behavior.

Importantly, the distinction between HAMN and mucinous adenocarcinoma hinges on the identification of destructive stromal invasion. Invasive glands elicit a fibroblastic, desmoplastic stromal response absent in non-invasive counterparts. Recognition of this invasion is crucial, as it defines the shift from “neoplasm” to “carcinoma” in the WHO framework.

#### 2.6.3. Molecular Features and Genetic Alterations

From a molecular standpoint, mucinous adenocarcinomas retain *KRAS* mutations in 60–90% of cases, while *GNAS* mutations become less frequent, reflecting a shift toward a more proliferative and less mucin-driven phenotype [29,31]. Enrichment of *TP53*, *SMAD4*, and APC/WNT alterations drives stromal invasion and cytologic aggressiveness [30,31]. A minority of cases harbor DNA repair defects including *ATM*, *BRCA2*, and mismatch repair deficiency (dMMR), with potential sensitivity to immune checkpoint inhibitors [31].

#### 2.6.4. Clinical Presentation, Prognosis, Management and Therapeutic Strategies

Clinically, mucinous adenocarcinomas of the appendix are often insidious in onset and discovered at advanced stages. Presentation may mimic acute appendicitis or manifest as an abdominal mass, ascites, or PMP [33]. Many cases are identified incidentally during surgery or imaging for unrelated conditions. The disease predominantly affects older adults, with a mean age at diagnosis between 65 and 70 years. In contrast to non-mucinous appendiceal adenocarcinomas, mucinous variants display a strong predilection for peritoneal dissemination, leading to extensive mucinous deposits within the abdominal cavity [30,32,33,34].

Although the pattern of spread is locally aggressive, the rate of systemic metastasis (hepatic or pulmonary) is comparatively lower, reflecting their mucin-secreting biology and peritoneal tropism. Prognosis depends on tumor grade, depth of invasion, nodal status, and completeness of cytoreduction. Reported 5-year overall survival ranges from 45% to 55%, with improved outcomes in cases managed aggressively with complete cytoreductive surgery and HIPEC [35]. Signet-ring cell differentiation, nodal metastases, and incomplete resection are the strongest predictors of poor outcome.

Compared with non-mucinous adenocarcinomas, mucinous variants tend to progress more slowly but are diagnosed later, often when peritoneal involvement is already established. Consequently, early recognition and accurate pathologic staging are pivotal for improving survival [30,32]. The cornerstone of treatment for mucinous adenocarcinoma of the appendix is complete surgical resection with histologically negative margins. In localized disease, right hemicolectomy is recommended due to the relatively high incidence of lymph-node metastasis and the need for adequate oncologic clearance. In cases with peritoneal dissemination, management parallels that of PMP, with CRS followed by HIPEC as the preferred strategy [20,34]. The objective is to eliminate any remaining microscopic disease and prevent the accumulation of mucinous material. While HIPEC is well established for low-grade PMP, its benefit in invasive mucinous adenocarcinoma remains under active investigation, with outcomes depending heavily on disease burden and completeness of cytoreduction [20,34]. For patients with nodal or systemic metastases, systemic chemotherapy typically 5-fluorouracil or oxaliplatin-based regimens modelled after colorectal cancer protocols is considered [32,33].

However, the rarity of the disease and its distinct biology mean that prospective, randomized data are lacking, and therapeutic decisions are often individualized based on multidisciplinary evaluation. Emerging molecular data suggest that targeted therapy or immunotherapy may become relevant for select subgroups, such as those harboring microsatellite instability (MSI-H) or *BRCA2*-mutated tumors, although evidence remains preliminary.

Taken together, these aspects highlight the prognostic factors, therapeutic controversies, and emerging molecular implications central to the management of mucinous appendiceal adenocarcinoma. Prognostically, tumor grade, completeness of cytoreduction, nodal involvement, and signet-ring cell differentiation remain the most consistent determinants of outcome [30,35]. From a therapeutic standpoint, important controversies persist systemic chemotherapy is largely extrapolated from colorectal protocols despite the distinct biology of these tumors, prospective randomized evidence is lacking, and the benefit of HIPEC in invasive mucinous adenocarcinoma, as well as the role of perioperative systemic chemotherapy, remains under active investigation and dependent on disease burden and completeness of cytoreduction [20,32,33,34]. From a molecular perspective, emerging biomarkers are beginning to acquire clinical relevance: MSI-high or dMMR tumors may be candidates for immune checkpoint inhibition, and *BRCA2* or other DNA-repair alterations may confer sensitivity to specific agents, although these approaches remain investigational and applicable only to small molecular subsets [31]. These considerations reinforce the need for molecular profiling and for treatment strategies developed specifically for appendiceal adenocarcinoma rather than extrapolated from colorectal cancer.

#### 2.6.5. Pathologic Staging and Risk Stratification

Accurate staging is critical for prognosis and therapeutic decision-making. According to the 8th edition of the AJCC Cancer Staging Manual, appendiceal mucinous adenocarcinomas are staged using a TNM system analogous to colorectal adenocarcinomas, but with specific considerations for appendiceal perforation and peritoneal spread [35].

T category reflects the depth of invasion through the appendiceal wall and into contiguous structures such as the cecum or ileum. N category denotes regional lymph-node metastases, which occur in up to 30% of cases. M category includes peritoneal dissemination or distant organ metastases, both of which carry grave prognostic significance.

Large population-based studies, such as those derived from the SEER database, have collected prognostic data incorporating tumor grade, lymph-node involvement, peritoneal dissemination, and patient age to refine risk stratification and guide adjuvant therapy decisions [12,33,35].

The optimal management of mucinous adenocarcinoma of the appendix requires multimodal integration combining precise histopathologic assessment, molecular profiling, and individualized surgical and systemic approaches to maximize survival and quality of life in this rare but challenging malignancy.

### 2.7. Signet Ring Cell Carcinoma (SRCC) and Poorly Differentiated Variants of the Appendix

#### 2.7.1. Definition and General Characteristics

Signet Ring Cell Carcinoma (SRCC) of the appendix represents one of the rarest and most biologically aggressive epithelial malignancies of the appendix, accounting for less than 1% of appendiceal neoplasms [36]. Histologically, it is defined by the predominance (>50%) of cells containing intracytoplasmic mucin that compresses the nucleus against the cell membrane, producing the hallmark “signet ring” morphology [7]. Unlike mucinous or conventional adenocarcinomas, SRCC and other poorly differentiated variants of appendiceal carcinoma exhibit marked cellular discohesion, diffuse infiltrative growth, and early dissemination, translating into an extremely poor prognosis. Appendiceal SRCC may arise de novo or through dedifferentiation of preexisting mucinous adenocarcinoma, with molecular and morphological evolution toward a high-grade, invasive phenotype [36,37]. Its clinical and biological behavior parallels that of gastric or colorectal SRCC, yet its molecular underpinnings are distinct, underscoring the importance of recognizing appendiceal origin through immunophenotypic and genomic profiling [9].

#### 2.7.2. Histopathologic Features

According to the 5th Edition of the WHO Classification (2019), appendiceal SRCC is defined as a carcinoma composed predominantly (>50%) of signet ring cells, although smaller foci of such morphology may coexist within conventional mucinous or poorly differentiated adenocarcinomas [7]. Histologically, SRCCs are composed of small, discohesive epithelial cells infiltrating the appendiceal wall in a diffuse, single-cell pattern, often extending into the subserosa and peri-appendiceal adipose tissue. This infiltrative pattern, coupled with extensive desmoplastic stromal reaction, explains the frequent transmural involvement and peritoneal seeding seen at presentation [37].

Tumor cells typically contain large mucin vacuoles, which displace the nuclei eccentrically, creating the eponymous “signet ring” appearance. Some variants show lesser mucin content and more solid architecture, reflecting partial glandular differentiation.

The background stroma may be densely fibrotic, hyalinized, or inflamed, and perineural and lympho-vascular invasion are almost universal [9]. Extracellular mucin may be present, though less abundant than in mucinous adenocarcinomas. Necrosis, stromal retraction, and lymphangitic permeation are common histologic clues to the diagnosis. Peritoneal carcinomatosis is a frequent finding, often discovered intraoperatively as widespread serous implants or gelatinous deposits that mimic pseudomyxoma peritonei but lack its indolent course [37].

Immunohistochemically, appendiceal SRCCs are typically CK20^+^, CDX2^+^, MUC2^+^, and CK7^−^, mirroring intestinal differentiation [7]. However, focal CK7 expression can occur, particularly in tumors with partial mucinous differentiation.

A pivotal diagnostic feature is the loss or marked reduction in E-cadherin expression, both by immunohistochemistry and at the molecular level, corresponding to loss of intercellular adhesion and accounting for the tumor’s diffuse and discohesive growth pattern. This E-cadherin downregulation distinguishes SRCC from more cohesive adenocarcinomas and correlates with the tumor’s aggressive biological behavior.

#### 2.7.3. Molecular Features and Genetic Alterations

Molecularly, SRCC of the appendix is distinct from LAMN and conventional mucinous adenocarcinomas, aligning more closely with poorly differentiated colorectal carcinomas [36,38]. *KRAS* and *GNAS* mutations, frequent in low-grade mucinous neoplasms, are infrequent or absent in SRCC, supporting a divergent tumorigenic pathway [36,38] while *TP53* and *SMAD4* mutations are significantly enriched, underscoring their role in promoting high-grade cytologic atypia, apoptosis resistance, and invasive potential [36].

CDH1 (E-cadherin) loss-of-function mutations, as well as promoter hypermethylation, are characteristic molecular events that disrupt adherent junctions and drive the epithelial–mesenchymal transition (EMT), the key mechanism underlying cellular lack of cohesion and invasiveness. Alterations in *PIK3CA*, *PTEN*, and *BRAF* occur sporadically, possibly defining minor molecular subsets amenable to targeted therapy [39]. A minority of cases exhibit MSI-H or dMMR, which, though rare, hold therapeutic relevance for immune checkpoint blockade [36]. Transcriptomic studies reveal activation of EMT-related transcription factors and upregulation of Wnt/β-catenin signaling, further reinforcing the link between dedifferentiation and invasiveness [40]. These findings suggest that SRCC arises through loss of epithelial polarity, acquisition of stem-like traits, and profound disruption of mucin-regulatory networks, leading to its unique morphology and lethal phenotype.

#### 2.7.4. Clinical Presentation, Prognosis, Management and Therapeutic Strategies

Clinically, appendiceal SRCC often presents at an advanced stage, frequently with diffuse peritoneal carcinomatosis or ovarian metastases in female patients. Common presenting symptoms include abdominal distension, ascites, bowel obstruction, or vague abdominal discomfort. Occasionally, SRCC masquerades as pseudomyxoma peritonei, yet the clinical course is much more aggressive, with rapid progression despite therapy [9,36]. Because of its insidious nature, diagnosis is often delayed and curative resection is rarely possible at presentation. Prognosis is uniformly poor, with reported 5-year overall survival rates between 6% and 14%, even under aggressive multimodal management [37,38]. Major adverse prognostic factors include transmural invasion (T4 stage), lymph-node metastasis, peritoneal or distant dissemination, poor differentiation/high-grade cytology and incomplete cytoreduction or positive margins [7,39]. Compared to mucinous adenocarcinoma, SRCC exhibits higher rates of hematogenous metastases, particularly to the liver, lungs, and bone, consistent with its loss of mucin-dependent adhesion and gain of migratory potential [7].

In multivariate analyses, signet ring morphology itself serves as an independent predictor of poor outcome, irrespective of tumor stage or treatment modality [9,38].

Due to the rarity of appendiceal SRCC, there is no consensus guideline, and treatment protocols are largely extrapolated from colorectal SRCC management strategies [35,37].

For localized disease, radical surgical resection (right hemicolectomy) with regional lymphadenectomy remains the mainstay, aiming for complete (R0) excision [37]. However, given the tumor’s diffuse infiltration and submucosal spread, complete resection is often challenging. In cases of peritoneal dissemination, CRS followed by HIPEC has been attempted. While this approach provides survival benefit in mucinous adenocarcinomas, its efficacy in SRCC is limited due to the tumor’s aggressive infiltration and poor chemosensitivity.

Systemic chemotherapy, typically based on FOLFOX (5-FU/oxaliplatin) or FOLFIRI (5-FU/irinotecan) regimens, is standard for advanced or node-positive disease [35,38]. Response rates remain modest, and median survival seldom exceeds 12–18 months in metastatic settings. Emerging evidence supports a potential role for immune checkpoint inhibitors in patients with dMMR or MSI-high SRCCs and for targeted therapies directed at PI3K/AKT/mTOR or *BRAF* pathways in specific molecular subsets [39].

However, prospective trials are lacking, and outcomes remain poor despite multimodal therapy, underscoring the urgent need for novel systemic approaches and molecularly guided treatment stratification.

#### 2.7.5. Pathologic Staging and Diagnostic Considerations

Pathologic staging follows the AJCC 8th Edition TNM system, which is largely aligned with that used for appendiceal adenocarcinomas, although specific considerations apply to mucinous neoplasms [7,9]. Nevertheless, the presence and extent of signet ring morphology should be explicitly reported, as it independently predicts adverse prognosis and reduced survival regardless of stage [7]. A critical diagnostic challenge is distinguishing primary appendiceal SRCC from metastatic SRCC of gastric or colorectal origin.

Immunohistochemical panels incorporating CDX2, SATB2, MUC2, and MUC5AC in conjunction with clinical and radiologic correlation, are essential for accurate diagnosis.

In particular, the diagnostic distinction from metastatic signet ring cell carcinoma of gastric origin is critical, since the two are morphologically indistinguishable but differ markedly in management and prognosis; an intestinal immunophenotype (CDX2^+^, SATB2^+^, CK20^+^) favors appendiceal origin, whereas CK7 positivity with CDX2/SATB2 negativity points toward an upper gastrointestinal primary, and clinical–radiologic correlation to exclude a gastric or colonic mass remains essential [7,9].

SATB2 positivity and co-expression of CDX2 strongly favor appendiceal or colorectal origin, while MUC5AC and CK7 positivity suggest possible gastric primary.

Ultimately, appendiceal SRCC represents a distinct clinicopathologic entity characterized by diffuse invasion, loss of cohesion, and catastrophic prognosis. Its recognition bears major implications for staging, prognosis, and emerging molecular therapies.

A comparative overview of the main mucinous and related epithelial neoplasms of the appendix, summarizing their cytological, molecular, and clinical features, is provided in Table 2.

### 2.8. Neuroendocrine Differentiation and Mixed Tumors of the Appendix

Neuroendocrine differentiation in appendiceal neoplasia encompasses a spectrum of histopathologic entities, from indolent well-differentiated neuroendocrine tumors (NETs) to mixed neuroendocrine non neuroendocrine neoplasms (MiNENs), which integrate glandular and neuroendocrine phenotypes within the same lesion. This heterogeneity mirrors the biological diversity of the appendix itself a site where epithelial, mucinous, and neuroendocrine lineages intersect.

While pure NETs represent the most frequent epithelial tumors of the appendix, their biological simplicity contrasts sharply with that of mixed neoplasms, where dual differentiation gives rise to unique diagnostic and therapeutic challenges [7,41].

The WHO (5th Edition, 2019) defines these entities based on the relative contribution and biological grade of their components, emphasizing that each compartment neuroendocrine and non-neuroendocrine must constitute at least 30% of the tumors for a diagnosis of MiNEN [41]. This refined taxonomy underscores a paradigm shift from morphology-based nomenclature to biologically and genetically informed classification, reflecting the growing understanding that appendiceal neoplasia is a continuum of cellular differentiation rather than a collection of discrete diseases.

#### 2.8.1. Neuroendocrine Tumors (NETs) of the Appendix

Well-differentiated appendiceal NETs are by far the most prevalent neoplasms of the appendix, accounting for up to 60–70% of appendiceal epithelial tumors [42]. They are most encountered as incidental findings during appendectomy for acute appendicitis, particularly in younger adults. These tumors originate from enterochromaffin (EC) cells located predominantly in the subepithelial layer of the distal third of the appendix, a region rich in serotonin-producing cells. Their neurosecretory activity and paracrine influence explain the stromal fibrosis and local tissue retraction often observed histologically.

Histologically, appendiceal NETs are well-circumscribed, submucosal nodules composed of uniform polygonal cells arranged in nests, ribbons, trabeculae, or rosettes [43].

The nuclei are round to oval, displaying finely stippled “salt-and-pepper” chromatin typical of neuroendocrine differentiation, while the cytoplasm is scant and eosinophilic. The overlying mucosa may exhibit fibrosis, ulceration, or retraction, attributed to local effects of serotonin and peptide hormone secretion. Invasion into the mesoappendix or lympho-vascular spaces is possible even in small lesions, although the overall biological behavior remains favorable.

Immunohistochemically, NETs demonstrate strong, diffuse expression of chromogranin A, synaptophysin, and CD56, confirming neuroendocrine differentiation. The Ki-67 proliferation index and mitotic rate are fundamental to grading according to WHO criteria (NET G1: Ki-67 < 3%; <2 mitoses per 10 HPF; NET G2: Ki-67 3–20%; 2–20 mitoses per 10 HPF; NET G3: Ki-67 > 20%; >20 mitoses per 10 HPF) [44].

The vast majority of appendiceal NETs are G1 tumors, correlating with indolent clinical behavior and excellent long-term survival [45]. However, higher-grade lesions though rare may exhibit increased mitotic activity, focal necrosis, and nuclear pleomorphism, features associated with greater metastatic potential.

Clinically, most appendiceal NETs are nonfunctional and discovered incidentally. Rarely, serotonin-secreting tumors may cause carcinoid syndrome, though this requires extensive hepatic metastases and is exceedingly uncommon in appendiceal primaries [45]. Small NETs (<1 cm) confined to the appendix tip are adequately treated with appendectomy alone, as the risk of nodal spread is negligible [35].

Tumors exceeding 2 cm, or those showing mesoappendiceal invasion > 3 mm, base involvement, or lympho-vascular invasion, warrant right hemicolectomy for complete lymph node clearance and optimal staging [46]. Intermediate-sized lesions (1–2 cm) are evaluated on a case-by-case basis, integrating histologic features and surgical margins.

Overall, the prognosis of localized appendiceal NETs is excellent, with 5-year overall survival exceeding 95% [42,45]. Regional lymph node metastases occur in 10–20% of tumors > 2 cm, while hepatic spread remains exceptional. Postoperative follow-up includes serum chromogranin A and urinary 5-HIAA monitoring in selected cases, though their sensitivity is limited in nonfunctional NETs. Cross-sectional imaging is reserved for higher-risk tumors.

#### 2.8.2. Mixed Neuroendocrine–Non-Neuroendocrine Neoplasms (MiNENs)

The concept of MiNENs, introduced in the WHO 2017 and reaffirmed in the 2019 WHO classification, replaces the earlier terminology Mixed Adeno-neuroendocrine Carcinoma (MANEC) [41]. MiNENs of the appendix are exceptionally rare, constituting less than 5% of appendiceal tumors, and embody a biphasic neoplastic process characterized by the coexistence of glandular/mucinous and neuroendocrine carcinoma (NEC) components, each contributing ≥ 30% of the neoplasm [47]. This dual differentiation may occur through clonal divergence from a common progenitor, or via trans-differentiation within a preexisting mucinous carcinoma, reflecting the remarkable plasticity of appendiceal epithelial cells. Morphologically, the non-neuroendocrine component most frequently corresponds to a mucinous adenocarcinoma, HAMN, or signet ring cell carcinoma, whereas the neuroendocrine element is typically a poorly differentiated NEC, of either small-cell or large-cell type. The two components may be distinct and demarcated by fibrous septa, suggesting bi-clonal development, or intimately admixed, reflecting intratumoral evolution from a common clone. The neuroendocrine compartment usually displays high mitotic activity, necrosis, and nuclear modelling, while the glandular portion retains mucin production and glandular architecture.

From an immunohistochemical point of view, it has been demonstrated that in some cases there may be positivity for PD-L1 and miss-match repair loss/MSI-H [41]. Ki-67 proliferation indices often exceed 50–80% in the NEC component, underscoring its high-grade biology. Molecular analyses support a shared clonal origin for both components, with subsequent divergent differentiation pathways driving phenotypic heterogeneity [41]. Common trunk mutations include *KRAS*, *TP53*, and *SMAD4*, mutations that underpin early tumorigenesis. The neuroendocrine compartment often accumulates additional alterations in *RB1*, *PTEN*, and *NOTCH* signaling pathways, contributing to dedifferentiation and proliferative autonomy [39]. In contrast, *GNAS* mutations hallmarks of mucinous neoplasms such as LAMN and HAMN are rarely detected in MiNENs, reinforcing their distinct molecular lineage. This genetic complexity translates into heterogeneous therapeutic responses, as the two compartments often differ markedly in chemosensitivity and growth kinetics, complicating treatment planning and prognostication.

Clinically, appendiceal MiNENs typically present at an advanced stage, manifesting as right lower quadrant mass, intestinal obstruction, or peritoneal carcinomatosis.

Radiologically and macroscopically, they may mimic mucinous adenocarcinoma or pseudomyxoma peritonei, yet the higher cellularity and proliferative rate of MiNENs portend a more aggressive course [41]. Prognosis is largely determined by the grade and extent of the neuroendocrine component. When this element is poorly differentiated, clinical behavior parallels small-cell carcinoma, with median survival below 2 years despite multimodal therapy [39]. Conversely, if the non-neuroendocrine component is low grade (LAMN), disease progression may be indolent, and long-term survival is possible after complete resection. The management of appendiceal MiNENs remains non-standardized and is guided by the dominant histologic component [41].

For adenocarcinoma-predominant tumors, treatment parallels colorectal cancer regimens, employing FOLFOX or FOLFIRI-based chemotherapy. When the neuroendocrine component dominates, platinum-based protocols (cisplatin/etoposide) akin to small-cell carcinoma therapy are preferred. Surgical resection remains pivotal: right hemicolectomy is the standard for localized disease, while cytoreductive surgery may be considered in cases of peritoneal dissemination, though supporting evidence remains anecdotal [48].

Overall, the neuroendocrine component carries decisive prognostic weight: a poorly differentiated, high-grade neuroendocrine element dictates an aggressive course comparable to small-cell carcinoma, whereas a low-grade non-neuroendocrine component allows a more indolent evolution. This dual biology represents the principal current treatment challenge, as the two compartments differ in chemosensitivity and no standardized regimen exists; management is therefore directed at the dominant and most aggressive component [39,41].

The advent of molecular profiling is reshaping therapeutic strategy; therefore, it is now essential to study MiNEN-specific somatic tumor mutations, as well as RNA sequencing data. These studies and data could potentially contribute to future clinical trials of MiNEN-specific therapies [41].

## 3. Molecular Pathogenesis and Genetic Landscape of Appendiceal Neoplasms

Understanding the molecular underpinnings of appendiceal mucinous neoplasms has transformed the way pathologists and clinicians conceptualize these tumors. Where once morphology alone guided classification and clinical decisions, multi-omics studies now reveal recurrent driver events, distinct molecular subgroups, and patterns of progression that parallel and in some ways diverge from those seen in colorectal cancer [31]. This molecular knowledge clarifies why lesions with deceptively indolent histology can nevertheless disseminate within the peritoneal cavity, and it creates opportunities for diagnostic precision and targeted interventions.

### 3.1. Recurrent Driver Mutations and Core Pathways

A consistent finding across genomic studies is the high prevalence of activating mutations in *KRAS* and frequent alterations in *GNAS*. These two events form a molecular hallmark of appendiceal mucinous neoplasia [31].

Activating alterations of *KRAS* (codons 12/13 most commonly) occur in the majority of LAMNs and remain frequent in HAMNs and mucinous adenocarcinomas. *KRAS* activation promotes proliferative signaling through MAPK and supports tumor survival and growth [12]. *GNAS* mutations, commonly at codon R201, are enriched in appendiceal mucinous tumors and drive mucin production through cAMP/PKA activation, leading to overexpression of mucin genes such as *MUC2* and *MUC5AC* [12,31]. The co-occurrence of *KRAS* and *GNAS* alterations is particularly characteristic, serving as a molecular signature that distinguishes appendiceal primaries from other mucinous neoplasms of the ovary or colon [12]. As the disease progresses from LAMN to HAMN and ultimately to mucinous adenocarcinoma, additional genetic events accumulate. Mutations in *TP53* and *SMAD4*, together with aberrations in APC/WNT signaling, are often implicated in this stepwise transformation, correlating with higher grade, invasive behavior, and poorer prognosis [12]. Other recurrent alterations include *RNF43*, *PIK3CA*, *ATM*, and *ERBB2* (HER2) amplifications, while *BRAF* mutations remain rare [31]. Copy-number gains and chromosomal instability increase with histologic grade, reflecting molecular progression toward malignancy.

The principal genetic alterations, their associated tumor subtypes, and their clinical significance are summarized in Table 3.

### 3.2. DNA Repair, MSI, and Tumor Mutational Features

Most appendiceal mucinous neoplasms are microsatellite stable (MSS); however, a small subset exhibits dMMR or MSI-H status. These cases, although rare, may benefit from immune checkpoint blockade, aligning with experiences in colorectal and gastric cancers [31]. Occasional tumors show defects in homologous recombination repair (HRD), including alterations in *BRCA2* or *ATM*, which could theoretically sensitize them to PARP inhibitors, though clinical validation remains limited [31]. Overall tumor mutational burden (TMB) is typically low in LAMNs, increasing in high-grade or invasive lesions.

### 3.3. Epigenetics, Methylation and Noncoding Drivers

Epigenetic dysregulation contributes to appendiceal tumorigenesis. Promoter hypermethylation of tumor suppressors and global methylation changes have been described, and altered expression of noncoding RNA (microRNA and long noncoding RNA) appears to influence mucin regulation, EMT programs, and immune interactions. Methylation profiles may ultimately help subclassify tumors and refine prognosis, although routine clinical application has not yet matured.

### 3.4. Transcriptomics and Phenotypic Programs

Transcriptomic analyses have identified biological programs associated with both mucin production and tumor progression in appendiceal mucinous neoplasms. Upregulation of MUC2, MUC5AC, and related secretory pathways represents a characteristic feature of mucinous tumors and appears closely linked to *GNAS* signaling [12]. In contrast, high-grade lesions show enrichment of EMT-related genes, matrix-remodeling enzymes, and stromal interaction pathways, supporting a more invasive phenotype. Moreover, the immune microenvironment varies from relatively “immune-cold” tumors to lesions enriched with macrophage-dominant inflammatory infiltrates, potentially influencing responsiveness to immunotherapy [49].

### 3.5. Tumor Microenvironment: Mucin, Stroma and Immune Contexture

The tumor microenvironment (TME) is central to the behavior and treatment response of appendiceal mucinous neoplasms [39,50]. Abundant extracellular mucin creates a biophysical barrier that shelters tumor cells from immune surveillance and impedes drug diffusion [50]. Mucin components themselves can sequester growth factors, alter immune cell trafficking, and promote peritoneal spread. Cancer-associated fibroblasts (CAFs) contribute to this process by producing collagen and hyaluronan, stiffening the stromal matrix and facilitating mucin retention [39]. The immune infiltrate typically consists of macrophages and suppressive myeloid cells, with limited cytotoxic T-cell activity, explaining the modest efficacy of immunotherapy in unselected PMP cases [49].

### 3.6. Clinical and Diagnostic Applications of Molecular Data

Molecular analysis has become essential for both diagnosis and treatment planning [12,31,35]. The concurrent presence of *KRAS* and *GNAS* mutations strongly supports an appendiceal origin for peritoneal mucinous disease of uncertain primary [12].

From a prognostic standpoint, the progressive accumulation of *TP53*, *SMAD4*, and WNT-pathway alterations parallels the transition to high-grade, invasive disease and correlates with worse outcomes, providing a molecular basis for prognostic stratification and for tailoring the aggressiveness of surgical and chemotherapeutic management [12].

In advanced disease, testing for MMR/MSI, *HER2* amplification, and rare actionable fusions is now recommended in international guidelines.

### 3.7. Therapeutic Implications and Targeted Strategies

Although direct *KRAS* targeting remains challenging, the advent of *KRAS* G12C inhibitors may be relevant for the small subset of cases harboring that mutation. MEK inhibitors have a theoretical rationale in *KRAS*-driven tumors, although clinical data remain limited [31]. Novel therapeutic directions include MAPK pathway inhibition through MEK inhibitors, which may have potential in *KRAS*-driven tumors despite sparse clinical data. Additionally, mucin and stromal targeting are being explored, involving enzymatic mucin degradation and hyaluronan modification to enhance intraperitoneal chemotherapy penetration [39,50]. Immunotherapy is currently reserved for MSI-H or high-TMB cases [39]. PARP inhibitors show potential use in HRD-positive tumors [31], while HER2-targeted therapy is aimed at ERBB2-amplified tumors. Antibody-drug conjugates and molecularly targeted agents are promising, particularly in preclinical testing as molecular characterization improves [39].

### 3.8. Liquid Biopsy, ctDNA and Monitoring

Circulating tumor DNA (ctDNA) and cell-free assays are being explored as non-invasive tools to detect residual disease, monitor recurrence after CRS/HIPEC, and profile actionable mutations when tissue is scarce [51]. Early studies show promise for longitudinal surveillance, though standardized thresholds and validation remain required.

### 3.9. Research Directions and Translational Models

Preclinical research is increasingly focused on organoid and patient-derived xenograft (PDX) models. These 3D systems recapitulate the architecture and molecular heterogeneity of appendiceal tumors, allowing drug sensitivity testing and exploration of mucin-targeting strategies [39,52]. Future directions include single-cell and spatial transcriptomics to dissect tumor–stroma–immune interactions, as well as clinical trials combining stromal modifiers, systemic targeted agents, and optimized intraperitoneal delivery. Ultimately, integration of molecular, transcriptomic, and TME data will enable a more refined, biology-driven classification of appendiceal neoplasms and pseudomyxoma peritonei.

## 4. Diagnosis

The diagnosis of AMNs and their peritoneal dissemination represents one of the most complex challenges in gastrointestinal oncology. These tumors often manifest insidiously, and their recognition requires a combination of clinical suspicion, radiologic accuracy, and meticulous pathologic evaluation. Because the biological spectrum of AMNs ranges from indolent low-grade lesions to invasive carcinomas, early and precise diagnosis is crucial to determine prognosis and guide optimal management strategies [8,28].

Clinically, appendiceal mucinous tumors frequently present with non-specific or subtle symptoms. Many patients initially complain of right lower quadrant pain, often mimicking acute appendicitis, while others report vague abdominal discomfort, bloating, or progressive distension. In a substantial proportion of cases, the diagnosis is incidental, made during imaging for unrelated conditions or during surgery for presumed appendicitis. A smaller subset of patients may present with advanced disease, characterized by abdominal distension, ascites, or symptoms of bowel obstruction secondary to peritoneal mucin accumulation, consistent with pseudomyxoma peritonei (PMP) [53]. Because of these variable presentations, clinicians must maintain a high index of suspicion when confronted with atypical or recurrent appendiceal symptoms, unexplained abdominal fluid collections, or right lower quadrant masses.

The initial diagnostic work-up includes routine laboratory investigations and tumor marker evaluation. Serum levels of carcinoembryonic antigen (CEA), carbohydrate antigen 19-9 (CA19-9), and cancer antigen 125 (CA125) are often elevated in patients with mucinous appendiceal tumors, particularly in those with peritoneal dissemination [54]. While these markers lack specificity, they are useful for postoperative surveillance and may reflect disease burden. Standard blood tests including complete blood count, liver and renal function, and nutritional indices are also obtained to assess baseline status and surgical risk [55].

Imaging plays a pivotal role in the diagnosis and staging of appendiceal mucinous neoplasms. Contrast-enhanced computed tomography (CT) of the abdomen and pelvis remains the cornerstone of preoperative assessment. On CT, these lesions appear as a well-encapsulated, tubular, low-attenuation mass arising from the cecum, occasionally demonstrating mural calcifications. The degree of appendiceal dilation may vary, and mural irregularity or wall discontinuity raises concern for rupture and peritoneal seeding. In the presence of peritoneal disease, CT may reveal low-density mucinous ascites, omental caking, and scalloping of visceral surfaces particularly the liver and spleen caused by compressive mucin accumulation [56,57]. The pattern and distribution of peritoneal involvement often follow the flow of peritoneal fluid, with predilection for dependent regions such as the pelvis, paracolic gutters, and subphrenic spaces [7].

Magnetic resonance imaging (MRI) provides superior soft tissue contrast and may be employed as a complementary modality, particularly for detailed evaluation of pelvic disease or when CT findings are equivocal [58]. On T2-weighted MRI, mucinous deposits typically demonstrate hyperintense signal intensity, and diffusion-weighted imaging can help differentiate mucinous from serous collections. MRI is also preferred for assessing small-volume disease, evaluating mesenteric involvement, and distinguishing acellular from cellular mucin deposits parameters with important prognostic implications [28]. In selected high-grade or mixed-histology cases, fluorodeoxyglucose positron emission tomography (FDG-PET/CT) may detect metabolically active foci, though most low-grade mucinous lesions are poorly FDG-avid [8].

A fundamental diagnostic principle is the strict avoidance of tumors rupture or iatrogenic dissemination. Percutaneous or transabdominal biopsy of a suspected appendiceal mucinous lesion is contraindicated, as even a fine-needle puncture may result in peritoneal seeding of mucinous epithelium, effectively converting a localized lesion into disseminated disease [55]. Similarly, laparoscopic manipulation of a distended appendix should be performed with extreme caution, ideally using an endoscopic retrieval bag for specimen extraction. When peritoneal implants are present, however, targeted biopsy of nodules or mucinous deposits is safe and may provide the histologic confirmation needed for diagnosis [53].

Histopathologic examination remains the diagnostic gold standard. The WHO and PSOGI guidelines have introduced standardized terminology and reporting criteria that address prior inconsistencies in nomenclature [7]. Pathologists are advised to evaluate the appendiceal wall architecture, cytologic grade, presence or absence of destructive stromal invasion, and extent of extra-appendiceal mucin whether acellular or containing neoplastic epithelium. Margin status, lympho-vascular and perineural invasion, and the presence of perforation or mural rupture are additional essential parameters. The histologic diagnosis LAMN, HAMN, or mucinous adenocarcinoma should be rendered in accordance with the WHO–PSOGI classification, ensuring reproducibility and prognostic relevance across institutions [7,8].

Intraoperatively, surgeons must handle the appendix as a potentially malignant structure. Accidental rupture can lead to dissemination of mucin and epithelial cells throughout the peritoneum, dramatically worsening prognosis [54]. For this reason, careful specimen orientation, use of impermeable retrieval bags, and detailed communication with the pathology team are mandatory. When peritoneal disease is present, intraoperative scoring systems such as the Peritoneal Cancer Index (PCI) and the Completeness of Cytoreduction (CC) score provide objective quantification of disease burden and surgical outcome, both of which are independent prognostic factors [57].

Once the diagnosis is established, disease staging is performed according to the AJCC 8th edition, which classifies peritoneal involvement as M1a (acellular mucin) or M1b (cellular mucin, further subdivided by grade) [56]. However, in mucinous neoplasms, peritoneal grade and cytologic atypia often outweigh traditional TNM criteria in predicting outcome. The PSOGI consensus therefore recommends integrating both systems in diagnostic reports for optimal clinical correlation [7,8].

After initial treatment, structured surveillance is essential given the risk of recurrence or progression to pseudomyxoma peritonei. Current international guidelines suggest follow-up for at least five years, typically involving serial CT scans every six months during the first two years, followed by annual imaging thereafter [53,58]. Tumor markers (CEA, CA19-9, CA125) should be measured at each visit and correlated with imaging findings. Patients with high-risk features such as extra-appendiceal cellular mucin, positive surgical margins, or wall perforation require closer monitoring. Conversely, patients with localized LAMN completely confined to the appendix and negative margins may be followed less intensively, often with imaging once per year. Recent studies have proposed risk-adapted surveillance protocols, though consensus on optimal frequency and modality remains limited [28]. Emerging evidence also suggests that ctDNA and liquid biopsy approaches could play a role in early detection of recurrence, though these technologies are not yet standard practice [51].

In summary, the diagnostic process for appendiceal mucinous neoplasms requires integration of clinical, radiologic, surgical, and pathologic data. Accurate diagnosis hinges upon multidisciplinary collaboration, adherence to consensus criteria, and meticulous handling of surgical specimens to prevent disease dissemination. Early recognition and correct classification not only guide appropriate treatment but also profoundly influence long-term prognosis, particularly in preventing or managing pseudomyxoma peritonei [28,53,54,55,56,57,58].

## 5. Pseudomyxoma Peritonei

AMNs encompass a spectrum of tumors ranging from LAMNs to more aggressive high-grade lesions. While many AMNs remain localized and indolent, a particularly significant and challenging complication is Pseudomyxoma Peritonei (PMP). This syndrome arises when mucin-secreting epithelial cells from a ruptured appendiceal tumor disseminate into the peritoneal cavity, producing copious gelatinous ascites. The pathophysiology of PMP reflects a unique combination of the relatively indolent behavior of low-grade neoplasms and the mechanical consequences of mucin accumulation, resulting in a condition that can extensively affect the peritoneal cavity without overtly aggressive tissue invasion [5,28].

### 5.1. Pathogenesis and Relationship to AMNs

The pathogenesis of PMP is intrinsically linked to the biology of AMNs. Low-grade appendiceal mucinous neoplasms have a propensity to perforate the appendiceal wall, either spontaneously or during minor trauma. When this occurs, neoplastic epithelial cells and mucin are released into the peritoneal cavity. Unlike conventional metastases, these cells do not typically invade organs deeply; instead, they implant on peritoneal surfaces and continue to secrete mucin. Over time, this leads to the classic gelatinous ascites and diffuse peritoneal involvement that define PMP [5,59].

Histopathologically, PMP is graded according to the cytologic characteristics of the disseminated cells. Low-grade disease is characterized by bland epithelial cells within abundant extracellular mucin, whereas high-grade disease exhibits significant atypia and mitotic activity, correlating with more aggressive clinical behavior and higher recurrence rates [58,59]. The intimate relationship between AMNs and PMP underscores the importance of early detection and careful pathological assessment of appendiceal tumors, as even seemingly indolent lesions may herald the onset of widespread peritoneal disease [5,28].

### 5.2. Clinical Presentation

The clinical presentation of Pseudomyxoma Peritonei is often subtle, reflecting the slow and insidious nature of the disease. Unlike aggressive malignancies, PMP rarely causes acute pain in its early stages [28,59]. Patients most commonly report a gradually increasing abdominal girth, which may be attributed to weight gain or bloating rather than a pathological process. Some may experience vague abdominal discomfort or a sense of heaviness that waxes and wanes, which can easily be overlooked or misattributed to gastrointestinal disturbances. Digestive symptoms are variable and often nonspecific. Early satiety, intermittent constipation, or changes in bowel habits may occur as mucin accumulates and compresses the intestines. Rarely, patients present with partial bowel obstruction, which can provide the first clue to an underlying pathology. In women, PMP can sometimes be misdiagnosed as an ovarian mass due to adnexal involvement. In many cases, PMP is discovered incidentally during imaging or surgical procedures performed for unrelated conditions, such as suspected appendicitis or gynecological problems. The insidious course of PMP is closely tied to its pathophysiology. Because mucin-secreting tumor cells implant on peritoneal surfaces without deeply invading tissues, the disease can spread extensively before causing significant symptoms. This delayed clinical recognition is one of the reasons PMP often presents at an advanced stage, despite originating from tumors that may have otherwise behaved indolently [28,60].

### 5.3. Diagnostic Evaluation

Diagnosing PMP requires a thoughtful combination of imaging studies, laboratory assessments, and histopathologic confirmation. The initial suspicion often arises from imaging, which provides clues to the characteristic appearance of mucinous ascites. CT is the modality of choice, frequently revealing low-attenuation, gelatinous material within the peritoneal cavity, often layered along peritoneal surfaces. A classic finding is the “scalloping” of solid organs, such as the liver or spleen, caused by the pressure of the accumulating mucin. CT can also detect localized or diffuse peritoneal implants, which may guide surgical planning [5,59,60]. MRI can complement CT, particularly when assessing the pelvis or complex areas where mucin collections may be difficult to delineate. MRI’s superior soft-tissue contrast can help distinguish mucinous material from fluid or other ascites, aiding in the assessment of disease distribution and surgical resectability.

Laboratory evaluation can support the clinical suspicion but is rarely definitive. Tumor markers such as CEA, CA 19-9, and CA-125 may be elevated, reflecting the mucinous tumor burden, but they lack specificity. Therefore, while useful for monitoring disease progression or recurrence, tumor markers alone cannot establish the diagnosis.

The definitive diagnosis of PMP relies on histopathology obtained through laparoscopic sampling or analysis of surgical specimens; percutaneous biopsy is contraindicated due to the risk of peritoneal seeding. Pathologists identify pools of extracellular mucin containing strips or clusters of mucinous epithelia, and histologic grading—low versus high—is critical for prognosis and therapeutic decision-making [59,60].

A key challenge in diagnosis is the indolent nature of PMP and the often-subtle early findings. Patients may have extensive peritoneal spread before overt symptoms appear, and radiologic findings can sometimes be mistaken for benign ascites or ovarian pathology. Thus, a high index of suspicion, particularly in patients with a history of appendiceal mucinous neoplasm, is essential for early recognition and effective management.

### 5.4. Management Strategies

The primary treatment for PMP is surgical, focusing on CRS in conjunction with HIPEC, which is the current standard of care for eligible patients. This approach achieves maximal cytoreduction while targeting microscopic residual disease with intraperitoneal chemotherapy, yielding five-year survival rates far superior to historical debulking strategies [5,59,60]. Even with optimal therapy, recurrence remains possible, necessitating structured long-term surveillance with imaging and tumor markers [28,61].

### 5.5. Prognosis

The prognosis of PMP is influenced by several interrelated factors. The histologic grade plays a significant role; low-grade disease associated with LAMNs generally carries a favorable prognosis, whereas high-grade disease predicts poorer outcomes [59,60].

The extent of the disease is assessed using the Peritoneal Cancer Index (PCI), with higher scores correlating with lower survival rates [5,60]. Additionally, the completeness of cytoreduction is the strongest predictor of long-term survival, as complete removal of visible disease is crucial [28,61]. If left untreated, the progressive accumulation of mucin can eventually lead to bowel obstruction, nutritional compromise, and morbidity, highlighting the clinical importance of early recognition and intervention [28,59].

## 6. Treatment of Appendiceal Mucinous Neoplasms (AMN) and Pseudomyxoma Peritonei (PMP)

The management of AMN and their peritoneal dissemination, known as PMP, represents one of the most complex and debated challenges in oncologic surgery. These diseases, though rare, display unique biological and clinical behaviors that distinguish them from other gastrointestinal malignancies. Treatment must therefore balance radical oncologic control with preservation of organ function and quality of life. The therapeutic paradigm for AMNs and PMP has undergone a dramatic evolution over the past decades. Historically considered incurable and managed with repeated debulking surgeries or palliative drainage, PMP now benefits from highly specialized multimodal treatment integrating CRS and HIPEC. This combined approach, pioneered in the late 20th century, has profoundly changed the natural history of the disease, offering survival rates once thought unattainable [54,62].

### 6.1. Surgical Management of Localized AMNs

For appendiceal mucinous neoplasms confined to the appendix, the cornerstone of treatment remains complete surgical excision. The type of resection is tailored according to the lesion’s histologic grade and anatomical extent. A simple appendectomy is adequate for well-localized low-grade lesions without perforation or extra-appendiceal mucin [8]. Conversely, right hemicolectomy may be indicated when there is base involvement, submucosal invasion, lympho-vascular spread, or invasive adenocarcinoma. The objective is to achieve negative margins while allowing accurate lymph node staging.

Intraoperative vigilance is essential. Discovery of mucinous deposits within the peritoneal cavity, particularly gelatinous or acellular mucin, raises suspicion for early PMP and necessitates broader surgical planning and peritoneal inspection [63]. In such scenarios, patients should ideally be referred to high-volume centers specialized in peritoneal surface malignancies.

### 6.2. Cytoreductive Surgery (CRS)

Once peritoneal spread occurs, cytoreductive surgery becomes the essential curative step. The procedure aims to remove all macroscopic disease by performing systematic peritonectomy and, when required, organ resections such as omentectomy, splenectomy, or resection of involved intestinal segments [54,64]. The PCI, introduced by Sugarbaker, remains a vital tool to quantify tumor burden across 13 abdominopelvic regions. Higher PCI scores correlate with poorer prognosis and reduced likelihood of complete cytoreduction [64]. The Completeness of Cytoreduction (CC) score ranging from CC-0 (no visible disease) to CC-3 (residual nodules > 2.5 cm) is one of the most powerful prognostic indicators. Only patients achieving CC-0 or CC-1 resection (residual disease < 2.5 mm) derive significant survival benefit [62]. Cytoreductive surgery is an extensive procedure, often lasting over ten hours and associated with considerable physiological stress. However, in experienced centers, perioperative mortality has dropped below 3%, while long-term survival for low-grade PMP exceeds 80% at five years [54]. These results are unprecedented when compared with traditional debulking surgery, which historically offered median survival of less than two years.

### 6.3. Hyperthermic Intraperitoneal Chemotherapy (HIPEC) and Pressurized Intraperitoneal Aerosol Chemotherapy (PIPAC)

The concept of delivering chemotherapy directly into the peritoneal cavity dates to the late 1970s, when surgeons first recognized that intraperitoneal administration could achieve high local drug concentrations with limited systemic toxicity. The addition of hyperthermia heating the perfusate to approximately 42 °C was introduced in the early 1980s by Spratt and later refined by Sugarbaker, who integrated HIPEC into the treatment algorithm for PMP [65,66]. This innovation marked a pivotal shift from purely surgical management to a true multimodal oncologic approach.

The rationale for HIPEC lies in several pharmacologic and biological principles. The peritoneal plasma barrier allows cytotoxic agents to remain in the peritoneal cavity at concentrations 20–100 times higher than systemic levels, thereby maximizing local tumoricidal activity while minimizing systemic exposure [66]. Hyperthermia itself exerts a synergistic effect: at temperatures between 41 °C and 43 °C, cell membranes become more permeable, facilitating drug penetration into tumor tissue. Moreover, heat induces direct apoptosis, denatures structural proteins, and potentiates the cytotoxic effects of several chemotherapeutic agents, particularly alkylating agents and platinum compounds [28,67]. During HIPEC, following completion of cytoreductive surgery, the abdomen is perfused with a heated chemotherapeutic solution for 60 to 90 min. Two main delivery techniques are currently used: the open technique, which allows manual stirring and temperature monitoring, and the closed technique, in which the abdomen is temporarily sealed to maintain pressure and reduce heat loss [65,67]. Perfusion flow rates and temperature are closely controlled to ensure homogeneous distribution of the chemotherapeutic solution throughout the peritoneal cavity. Commonly used agents include mitomycin C, historically the standard for low-grade PMP; oxaliplatin, often in combination with intravenous 5-fluorouracil and leucovorin for high-grade disease; and cisplatin-doxorubicin combinations adopted in several Asian centers [68,69]. Despite regional variations, the efficacy of HIPEC appears more dependent on the completeness of cytoreduction than on the specific drug regimen used [28]. The clinical impact of HIPEC has been transformative. Multiple retrospective studies, registry analyses, and international consensus statements support CRS-HIPEC as the preferred treatment strategy for PMP, although high-level randomized evidence remain limited because of the rarity of the disease. Five-year survival rates now reach 70–90% for low-grade PMP and 40–60% for high-grade disease, depending on the extent of resection and histopathologic features [28,54,62]. Moreover, disease-free survival and quality of life are significantly improved, with many patients resuming normal activities after full recovery.

Notably, the PSOGI and subsequent consensus statements from the Chicago and Lyon meetings have recognized CRS-HIPEC as standard treatment for PMP management [70]. These guidelines emphasize the need for treatment in high-volume, specialized centers where multidisciplinary expertise ensures optimal patient selection, surgical precision, and postoperative care.

From a mechanistic standpoint, HIPEC represents more than a local chemotherapeutic procedure it acts as an intraoperative adjuvant therapy targeting microscopic residual disease. Its effectiveness depends not only on pharmacologic potency but also on meticulous temperature control, drug kinetics, and the ability to achieve uniform exposure of all peritoneal surfaces.

Nevertheless, HIPEC is not without limitations. The procedure entails significant morbidity, including risks of infection, anastomotic leak, renal toxicity, and hematologic suppression [54]. Furthermore, the absence of randomized phase III trials comparing CRS-HIPEC to modern systemic therapy has led to ongoing debate regarding its absolute survival benefit. However, given the rarity of PMP, accumulating high-level evidence remains challenging, and observational data continue to support its clinical superiority [28,54,70]. A more critical appraisal of the available evidence is also warranted regarding the heterogeneity of HIPEC protocols and the choice of intraperitoneal agent. Despite decades of clinical use, no universal consensus exists on the optimal HIPEC parameters, including drug selection, dose, temperature, and perfusion duration, and this methodological heterogeneity limits the comparability of published series. The two most widely used agents, mitomycin C and oxaliplatin, have been directly compared in appendiceal disease, with randomized data indicating broadly comparable long-term survival, while oxaliplatin-based regimens have been associated with higher rates of perioperative complications [68]. Treatment outcomes are also strongly influenced by case volume and center experience, and current recommendations rest largely on retrospective data and expert consensus rather than high-level evidence [67]. These considerations underscore the need for cautious, individualized application and for prospective studies specific to appendiceal disease.

In this context, the results of the randomized phase III PRODIGE-7 trial, although conducted in colorectal rather than appendiceal peritoneal metastases, have significantly influenced the perception of the role of HIPEC. The trial found no overall survival benefit from the addition of short-duration oxaliplatin-based HIPEC to complete cytoreductive surgery, while perioperative morbidity was higher in the HIPEC arm, highlighting the predominant prognostic role of complete cytoreduction itself [71]. These findings prompted a critical reassessment of HIPEC in colorectal disease and fueled the broader debate on its added value. It must be emphasized, however, that these results are not directly transferable to PMP and appendiceal mucinous neoplasms, whose distinct mucinous biology, indolent course, and different chemosensitivity continue to support a central role for CRS-HIPEC in this specific setting.

Recent refinements in technique such as improved perfusion systems, use of closed-circuit monitoring, and enhanced recovery protocols have further reduced perioperative complications. In parallel with these technical advancements, Pressurized Intraperitoneal Aerosol Chemotherapy (PIPAC) has emerged as a novel minimally invasive approach designed to enhance drug delivery within the peritoneal cavity [72,73,74]. PIPAC delivers chemotherapy as a fine aerosol under controlled pressure through a laparoscopic system. This pressurization promotes a more uniform spatial distribution of the drug and facilitates deeper tissue penetration, potentially improving cytotoxic efficacy against peritoneal tumor nodules. The procedure can be performed repeatedly at defined intervals, allowing for iterative local therapy in patients with recurrent or non-resectable PMP and other peritoneal surface malignancies.

Despite this promise, the current evidence supporting PIPAC must be interpreted with caution. Available data derive predominantly from phase I and II studies and small, heterogeneous patient cohorts, often with short follow-up, and convincing evidence regarding its impact on overall survival is still lacking. No randomized trial has yet defined its role relative to established or palliative strategies in PMP. Consequently, the precise place of PIPAC within current treatment algorithms remains uncertain, and its use should at present be regarded as investigational and confined to specialized centers and prospective protocols, pending higher-level evidence from ongoing and future studies [73,74].

**Figure 3:**
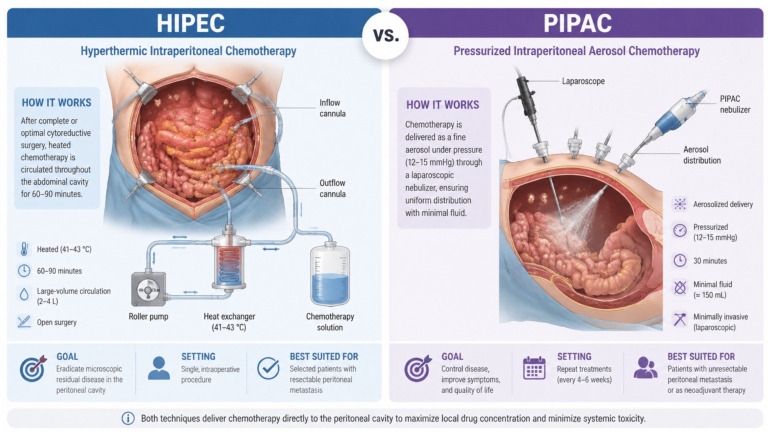
Comparative overview of HIPEC and PIPAC in the management of peritoneal metastases. A schematic comparison between HIPEC and PIPAC is presented. HIPEC entails intraoperative circulation of heated chemotherapy within the abdominal cavity following cytoreductive surgery, with the objective of eliminating microscopic residual disease. Conversely, PIPAC delivers aerosolized chemotherapy laparoscopically under pressure, enabling repeated minimally invasive treatments in patients with unresectable or advanced peritoneal disease. The figure elucidates distinctions in drug delivery modality, procedural setting, therapeutic objectives, and clinical indications. This figure is an original creation by the authors and has not been reproduced or adapted from previously published sources.

The main technical principles and clinical characteristics of HIPEC and PIPAC are compared in Table 4 and Figure 3.

### 6.4. Systemic and Emerging Therapies

While CRS-HIPEC remains the treatment of choice for resectable disease, systemic chemotherapy retains a role in non-operable, recurrent, or high-grade cases. Regimens based on fluoropyrimidines (FOLFOX or FOLFIRI) are used by analogy with colorectal cancer, although response rates are modest [77,78].

In recent years, the integration of molecular profiling has unveiled potential therapeutic targets. Recurrent mutations in *KRAS*, *GNAS*, and *PIK3CA*, along with less frequent alterations in *TP53* and *SMAD4*, have been identified in appendiceal mucinous tumors [9].

Furthermore, immunotherapy represents a promising frontier. Although most PMP cases exhibit low immunogenicity, a minority demonstrate MSI-H or high tumor mutational burden (TMB-H), making them candidates for immune checkpoint inhibitors. Ongoing clinical trials are exploring these strategies in combination with regional therapy to overcome the mucinous barrier that limits drug penetration. The treatment of appendiceal mucinous neoplasms and Pseudomyxoma Peritonei has evolved from palliation to precision surgery and targeted therapy. The integration of cytoreductive surgery and HIPEC has revolutionized outcomes, transforming a once-terminal condition into a potentially curable disease for many patients. While CRS-HIPEC remains the cornerstone of therapy, the future lies in refining patient selection through molecular profiling, integrating novel intraperitoneal techniques such as PIPAC, and harnessing systemic and immunologic approaches to further improve survival and quality of life. A multidisciplinary, patient-centered approach implemented within specialized centers remains the most effective model of care for these rare and complex diseases.

## 7. Discussion and Future Perspectives

The management of AMN and PMP has undergone a remarkable transformation over recent decades. From a once uniformly fatal disease characterized by relentless mucin accumulation within the peritoneal cavity, it has evolved into a complex but potentially controllable condition through multidisciplinary approaches.

CRS combined with HIPEC has transformed the prognosis of PMP, achieving five-year survival rates of 70–90% in low-grade disease. Despite these advances, recurrence continues to affect a substantial proportion of cases, particularly in high-grade variants, and the morbidity of extensive surgical procedures remains significant. The biological heterogeneity of these neoplasms and their unique mucinous microenvironment continue to limit the effectiveness of systemic therapies, underscoring the need for more targeted and individualized strategies [54,62,70].

Future progress in this field is likely to depend on a deeper understanding of tumor biology and the development of translational models that faithfully reproduce the intricate features of these diseases. In this regard, one of the most promising frontiers in recent years has been the advent of three-dimensional (3D) organoid cultures, which have opened new avenues for both research and clinical innovation. Organoids, derived from patient tumor samples or stem cells, are capable of self-organizing into miniature versions of the original tissue, preserving its structural, genetic, and functional characteristics. Unlike traditional two-dimensional cell lines, organoids maintain cellular heterogeneity, spatial organization, and the complex signaling dynamics that define tumor behavior in vivo [79,80]. The establishment of organoids derived from appendiceal mucinous tumors and PMP represents an important methodological advance, although its clinical translation remains at an early, investigational stage. For decades, researchers struggled to model these diseases due to the dominance of extracellular mucin and the difficulty of isolating viable tumor cells. Recent advances in culture techniques, using extracellular matrices such as Matrigel and specialized growth factor cocktails, now allow stable propagation of organoids that retain the hallmarks of PMP: abundant mucin secretion, glandular morphology, and expression of lineage markers such as MUC2, CK20, and CDX2 [76,81]. Importantly, these organoids also preserve the key genetic alterations characteristic of appendiceal mucinous neoplasms, including *KRAS* and *GNAS* mutations, which play central roles in mucin overproduction and peritoneal dissemination.

The potential applications of organoid technology in this context are wide-ranging and deeply transformative. From a research perspective, organoids provide an ideal system to study the biological mechanisms that drive tumor progression, mucin secretion, and resistance to therapy. They enable the dissection of how the tumor interacts with its microenvironment a particularly critical issue in PMP, where dense mucin and fibroinflammatory stroma create physical and biochemical barriers that impair drug delivery and foster immune evasion. From a clinical standpoint, organoids may eventually enable a more personalized approach to treatment. By testing various drugs directly on patient-derived organoids, clinicians can predict how an individual’s tumor will respond to specific agents, including HIPEC drugs such as mitomycin C or oxaliplatin. This approach, known as “functional precision oncology”, allows tailoring of therapy to the unique biology of each tumor, minimizing unnecessary toxicity while maximizing therapeutic benefit [82,83].

Moreover, organoids provide an unprecedented opportunity to explore new treatment modalities and refine existing ones. By simulating HIPEC conditions ex vivo replicating perfusion temperature, drug concentration, and exposure duration researchers can evaluate the pharmacodynamic and cytotoxic effects of intraperitoneal agents in a controlled yet physiologically relevant environment. Such studies may elucidate why certain regimens perform better in low-grade versus high-grade disease, or how mucin density affects chemotherapy penetration and efficacy [75]. This knowledge could guide optimization of HIPEC protocols, identifying the most effective combinations and improving outcomes across disease subtypes.

Another compelling application lies in the integration of organoids with other cellular components to recreate the tumor microenvironment more comprehensively. Co-culture systems combining organoids with immune cells or cancer-associated fibroblasts are now being developed to study immune–tumor interactions, cytokine signaling, and stromal modulation [84,85]. These complex models allow evaluation of immunotherapeutic strategies such as checkpoint inhibitors or CAR-T cells within a realistic biological context, something previously unachievable in PMP research. Furthermore, genetic engineering tools like CRISPR/Cas9 can be applied to organoids to model key molecular events, investigate the role of specific mutations, and identify potential therapeutic vulnerabilities [86].

The implications of these advancements extend beyond laboratory research. In clinical settings, the integration of organoid-based testing into multidisciplinary decision-making is becoming increasingly feasible. Some cancer centers already employ patient-derived organoids to support therapeutic choices in gastrointestinal malignancies, generating personalized drug sensitivity profiles within clinically relevant timeframes [82]. For PMP, this approach represents a promising preclinical tool that may, in the future, assist treatment planning. For instance, by helping to identify patients more likely to respond to oxaliplatin-based HIPEC versus those better suited for mitomycin C, although this application remains investigational and awaits prospective validation. International collaborations such as the Human Cancer Models Initiative (HCMI) and emerging PMP-specific organoid biobanks are laying the groundwork for standardized repositories of patient-derived models, which will facilitate reproducibility, large-scale screening, and collaborative translational research. It should be emphasized, however, that whereas CRS-HIPEC constitutes the established standard of care, organoid-guided therapy remains an experimental approach that has not yet entered routine clinical practice.

Nevertheless, significant challenges remain before organoid models can be fully integrated into clinical workflows. Technical variability, differences in culture success rates, the lack of vascularization, and limited immune representation all constrain their predictive capacity. Establishing organoids is still resource-intensive and time-consuming, posing logistical barriers for real-time clinical application. Yet, rapid progress in microfluidic “organ-on-chip” platforms, 3D bioprinting, and advanced co-culture techniques is addressing many of these limitations, bringing organoids closer to recapitulating the true tumor microenvironment [87,88]. As these technologies evolve, they promise to refine preclinical testing, accelerate drug discovery, and enable truly individualized treatment strategies for peritoneal surface malignancies.

Taken together, these developments suggest that the future management of appendiceal mucinous neoplasms and Pseudomyxoma Peritonei will be shaped by the convergence of molecular biology, translational modelling, and precision medicine, with organoid technology occupying a prominent role alongside the established foundation of CRS and HIPEC. These prospects, however, must be weighed against several unresolved controversies and methodological limitations that temper current enthusiasm.

### Controversies and Limitations

Despite the progress outlined above, a balanced appraisal of the evidence base is warranted across three areas in which enthusiasm has, at times, outpaced validation: the methodological quality of the data supporting CRS-HIPEC, the still-preliminary status of PIPAC, and the translational gap separating organoid models from clinical application in PMP.

The survival benefits attributed to CRS-HIPEC derive predominantly from retrospective registry analyses, large institutional cohorts, and international consensus statements rather than from phase III randomized trials comparing CRS-HIPEC with modern systemic therapy [28,54,62]. The rarity of PMP has historically precluded adequately powered prospective trials, a limitation explicitly recognized in both the PSOGI and Chicago Consensus documents [8,70]. This evidentiary structure carries two consequences that reviews seldom emphasize. First, the selection bias intrinsic to registry data, in which treatment is largely confined to fit patients managed in high-volume specialized centers, likely contributes to the favorable survival figures of 70–90% reported in low-grade disease, as illustrated by the large series of Chua et al. [54] and Kusamura et al. [67]. These outcomes may not be reproducible in lower-volume or non-specialist settings, where patient selection and surgical expertise are less standardized. Second, the morbidity of an extensive procedure raises a legitimate concern regarding overuse in patients unlikely to achieve complete cytoreduction. None of this invalidates the role of CRS-HIPEC, the consistency of benefit across independent cohorts constitutes meaningful, if observational, evidence but it argues for cautious interpretation of reported outcomes and for restricting the procedure to centers meeting recognized volume and quality standards.

PIPAC has attracted interest as a minimally invasive, repeatable option for unresectable or recurrent disease, yet its evidence base remains thin. The available data, including the multicenter experience of Alyami et al. [73] and the systematic review by Di Giorgio et al. [74], consist largely of retrospective analyses and single-arm feasibility studies from specialized units; no randomized trial has compared PIPAC with alternative palliative strategies or systemic chemotherapy in PMP or appendiceal mucinous neoplasms specifically. Reported radiological and cytological response rates of 50–70% rest on criteria whose prognostic value in mucin-dominant disease is incompletely validated, and aerosol tissue penetration of only 1–2 mm [73] casts doubt on efficacy against bulky or dense mucinous deposits. Because the reproducibility of these results outside expert environments has not been established, PIPAC in PMP is best regarded as an investigational approach that should be confined to prospective protocols in experienced centers until higher-level evidence accumulates.

A comparable gap separates organoid technology from clinical validation. The studies that have established organoid cultures from appendiceal mucinous neoplasms and PMP, such as that of Varinelli et al. [76], remain proof-of-concept or feasibility investigations involving small patient numbers, and no prospective study has yet validated concordance between organoid drug-sensitivity profiles and patient outcomes after HIPEC or systemic therapy in this disease specifically. The technical constraints noted earlier (variable culture success rates, absence of vascularization, limited stromal and immune representation, and substantial cost) are not peripheral caveats but fundamental limits on predictive validity and scalability. Although organoid-guided therapy has shown utility in metastatic gastrointestinal cancer [82] and other settings, extrapolation to PMP is premature without dedicated validation, and standardized protocols together with structured biobanking initiatives such as the Human Cancer Models Initiative remain prerequisites for meaningful translation. For the present, organoid-based functional precision oncology in PMP should be presented as a promising investigational strategy rather than an established clinical tool.

## 8. Conclusions

Appendiceal mucinous neoplasms and Pseudomyxoma Peritonei represent a distinctive clinical and biological entity within the spectrum of gastrointestinal malignancies. Their rarity, complex pathology, and peculiar dissemination patterns have long challenged clinicians and researchers alike. Over the past few decades, however, our understanding of these diseases has profoundly evolved, transforming what was once a uniformly fatal condition into a treatable, and in some cases, curable disease. This progress has been achieved through the convergence of surgical innovation, refined pathologic classification, and translational research.

The introduction of CRS combined with HIPEC stands as the cornerstone of this transformation. The concept, rooted in the pioneering work of Sugarbaker and others in the late twentieth century, revolutionized the management of peritoneal surface malignancies. CRS aims for the meticulous removal of all visible tumor deposits, while HIPEC targets microscopic residual disease through the direct administration of heated chemotherapeutic agents within the peritoneal cavity. This dual approach has produced unprecedented survival outcomes, particularly in patients with low-grade disease, and remains the standard of care in specialized centers worldwide. Despite its efficacy, CRS–HIPEC remains a demanding intervention, requiring careful patient selection and multidisciplinary expertise to balance survival benefit with procedural morbidity. Nonetheless, important limitations persist. Recurrence rates remain substantial, and therapeutic options for unresectable or recurrent disease are still limited. Conventional systemic chemotherapy has shown modest efficacy, reflecting the biological distinctiveness of appendiceal mucinous neoplasms and the pharmacologic barriers imposed by mucinous ascites. These challenges have catalyzed a renewed emphasis on molecular profiling, targeted therapies, and biologically informed treatment strategies. The elucidation of key genetic alterations such as mutations in *KRAS*, *GNAS*, and *TP53* has begun to shed light on the molecular drivers of mucin production and peritoneal dissemination, providing potential entry points for precision medicine.

In parallel with these established approaches, organoid technology has emerged as a promising translational tool. By enabling ex vivo testing of therapeutic agents on patient-derived models, organoids may, in the future, support more individualized treatment selection and the optimization of intraperitoneal regimens. At present, however, this remains an investigational approach that requires prospective validation before it can be incorporated into routine clinical practice.

In conclusion, the story of appendiceal mucinous neoplasms and Pseudomyxoma Peritonei exemplifies how sustained multidisciplinary collaboration and translational research can transform a once intractable condition into one increasingly understood and treatable. The integration of CRS–HIPEC with cutting-edge technologies such as organoid modelling and molecular profiling heralds a new phase in the evolution of this field, one characterized not only by improved survival but also by a more nuanced, patient-centered vision of care. The challenge that now remains is to consolidate these advances into reproducible, accessible, and evidence-based clinical pathways that can benefit all patients facing this rare yet formidable disease. From a clinical standpoint, the most immediate priorities are to refine patient selection for CRS-HIPEC, to standardize the management of recurrent and unresectable disease, and to incorporate molecular profiling into routine decision-making. For future research, the key directions include the prospective validation of organoid-based functional testing in PMP, the conduct of multi-institutional studies to strengthen the evidence base for emerging intraperitoneal and targeted therapies, and the establishment of shared biobanks and collaborative networks to overcome the limitations imposed by the rarity of these diseases.

## Figures and Tables

**Table 1:** Evolution of the terminology and classification of appendiceal mucinous neoplasms.

Historical/Obsolete Term	Current Terminology (WHO 2019/PSOGI 2016)	Definition and Notes	Reason for Revision
Mucocele	Not a diagnostic entity (descriptive term only)	Generic term for any mucin-filled cystic dilatation of the appendix	Failed to distinguish non-neoplastic processes from true neoplasms capable of peritoneal dissemination
Mucinous cystadenoma	Low-Grade Appendiceal Mucinous Neoplasm (LAMN)	Mucinous neoplasm with low-grade cytologic atypia and pushing, non-infiltrative growth	Replaced to reflect the biological potential for peritoneal spread despite a “benign” appearance
Mucinous cystadenocarcinoma	High-Grade Appendiceal Mucinous Neoplasm (HAMN)/Mucinous adenocarcinoma	High-grade cytologic atypia without (HAMN) or with (adenocarcinoma) infiltrative invasion	Old term conflated non-invasive high-grade lesions with frankly invasive carcinoma
Mucosal hyperplasia	Non-neoplastic (reactive) change	Hyperplastic mucinous epithelium without neoplastic features	Clarified as a non-neoplastic process, distinct from true neoplasia

The modern classification, jointly shaped by the WHO (5th Edition, 2019), the PSOGI consensus (2016), and the AJCC staging system (8th Edition), emphasizes cytologic grade, pattern of invasion, and the cellularity of peritoneal mucin as the key determinants of biological behavior and prognosis.

**Table 2:** Comparative summary of the main mucinous and related epithelial neoplasms of the appendix.

Feature	LAMN	HAMN	Mucinous Adenocarcinoma	Signet Ring Cell Carcinoma (SRCC)
Cytologic grade	Low-grade atypia	High-grade atypia	High-grade atypia	High-grade, poorly differentiated
Pattern of invasion	Pushing (expansile), non-infiltrative	Pushing, non-infiltrative	Infiltrative, destructive stromal invasion	Diffuse, single-cell infiltrative growth
Architecture	Villous, flat or undulating mucinous epithelium	Complex, cribriform or micropapillary	Irregular glands with desmoplasia	Discohesive signet ring cells with intracytoplasmic mucin
Mucin	Abundant, extracellular	Abundant, extracellular	Variable, often with cellular pools	Predominantly intracytoplasmic
Key molecular alterations	*KRAS* (>90%), *GNAS* (~70%); *TP53* typically absent	*KRAS*, *GNAS* plus *TP53*, *SMAD4*, occasional WNT	*KRAS* (60–90%), *GNAS* less frequent; *TP53*, *SMAD4*, APC/WNT; rare ATM/*BRCA2*/MMR defects	*KRAS/GNAS* infrequent or absent; *TP53*, *SMAD4* enriched; CDH1 (E-cadherin) loss
Risk of peritoneal dissemination	Low if confined; rises after wall rupture	Higher than LAMN	High	High, with early diffuse dissemination
Associated peritoneal disease	Low-grade mucinous carcinoma peritonei (PMP)	Low- or high-grade PMP	High-grade mucinous carcinoma peritonei	High-grade peritoneal disease; also, hematogenous spread
Prognosis	Excellent if confined; favorable in low-grade PMP	Intermediate	Poorer, grade- and extent-dependent	Very poor (5-year OS ~6–14%)
Typical management	Appendectomy if confined; CRS/HIPEC if disseminated	Appendectomy to CRS/HIPEC according to extent	CRS/HIPEC ± systemic chemotherapy	CRS/HIPEC + systemic chemotherapy; guarded outcome

Abbreviations: LAMN, low-grade appendiceal mucinous neoplasm; HAMN, high-grade appendiceal mucinous neoplasm; SRCC, signet ring cell carcinoma; PMP, pseudomyxoma peritonei; CRS, cytoreductive surgery; HIPEC, hyperthermic intraperitoneal chemotherapy; OS, overall survival.

**Table 3:** Key genetic alterations in appendiceal neoplasms, associated tumor subtypes, and clinical significance.

Genetic Alteration	Associated Tumor Subtype(s)	Clinical Significance
*KRAS*	LAMN, HAMN, mucinous adenocarcinoma	Drives proliferative signaling;
*GNAS*	LAMN, HAMN	Drives mucin hypersecretion; helps distinguish appendiceal from colorectal/ovarian primaries; linked to treatment resistance
*TP53/SMAD4*	HAMN, mucinous adenocarcinoma, SRCC, MiNEN	Mark progression to high-grade, invasive disease; adverse prognosis
*CDH1* (E-cadherin) loss	SRCC	Loss of cell adhesion; diffuse infiltrative, aggressive growth
*RB1/PTEN/NOTCH*	Neuroendocrine component of MiNEN	Neuroendocrine dedifferentiation; aggressive behavior
MMR deficiency/MSI-high	Subset of adenocarcinoma, SRCC, MiNEN	Potential sensitivity to immune checkpoint inhibitors

**Table 4:** Comparison of HIPEC and PIPAC: technical principles and clinical characteristics.

Characteristic	HIPEC	PIPAC	References
Therapeutic principle	Administration of heated liquid chemotherapy (≈42 °C) directly into the peritoneal cavity after complete cytoreductive surgery (CRS) to eradicate residual microscopic disease.	Administration of aerosolized chemotherapy under pressure into the peritoneal cavity to enhance tissue penetration in non-resectable or recurrent peritoneal disease.	Spratt J.S. et al., 1980 [65]; Sugarbaker P.H., 1995 [66]; Solass W. et al., 2013 [72]; Alyami M. et al., 2021 [73]
Technical approach	Performed intraoperatively immediately after CRS; the heated perfusate circulates for 60–90 min at 41–43 °C using a closed or open (“coliseum”) system.	Minimally invasive laparoscopic procedure: chemotherapy is nebulized under pressure (≈12–15 mmHg) into the abdominal cavity for 30 min, usually repeated in cycles.	Kusamura S. et al., 2021 [67]; Solass W. et al., 2013 [72]; Alyami M. et al., 2021 [73]
Common chemotherapeutic agents	Mitomycin C, Oxaliplatin (often with 5-FU and leucovorin for high-grade forms), Cisplatin ±.	Cisplatin, Doxorubicin, or Oxaliplatin in aerosolized form; lower doses used to minimize systemic toxicity.	Levine E.A. et al., 2014 [68]; Zhuang X. et al., 2022 [69]; Solass W. et al., 2013 [72]; Alyami M. et al., 2021 [73]
Temperature	41–43 °C (controlled hyperthermia provides synergistic cytotoxicity and increases membrane permeability).	Normothermic or mildly hyperthermic (36–39 °C); main effect is pressure-driven rather than temperature-dependent.	Sugarbaker P.H., 1995 [66]; Solass W. et al., 2013 [72]
Duration	Continuous perfusion for 60–90 min.	Approximately 30 min per cycle; procedures repeated every 4–6 weeks.	Spratt J.S. et al., 1980 [65]; Solass W. et al., 2013 [72]
Main indications	Resectable PMP, appendiceal mucinous neoplasms after CRS and primary tumors (mesothelioma, papillary serous); selected colorectal, gastric, or ovarian peritoneal metastases.	Non-resectable or recurrent PMP and peritoneal metastases; palliative or “bridge” therapy before CRS-HIPEC and bidirectional treatment in combination with systemic chemotherapy.	Kusamura S. et al., 2021 [67]; Alyami M. et al., 2021 [73]
Main advantages	High local drug concentration (20–100× systemic levels); synergistic effect of hyperthermia.	Minimally invasive and repeatable procedure with reduced morbidity; enables locoregional drug delivery in non-surgical candidates.	Chua T.C. et al., 2012 [54]; Kusamura S. et al., 2021 [67]; Solass W. et al., 2013 [72]; Alyami M. et al., 2021 [73]
Main limitations	Significant morbidity (infection, anastomotic leak, nephrotoxicity); prolonged operative time; requires specialized high-volume centers.	Limited tissue penetration (~1–2 mm); preliminary clinical data; lack of randomized controlled trials directly comparing with HIPEC.	Levine E.A. et al., 2014 [68]; Kusamura S. et al., 2021 [67]; Solass W. et al., 2013 [72]; Alyami M. et al., 2021 [73]
Reported clinical outcomes	5-year overall survival: 70–90% in low-grade PMP; 40–60% in high-grade disease.	Radiologic and cytologic responses in 50–70% of selected cases; may allow secondary conversion to CRS-HIPEC.	Chua T.C. et al., 2012 [54]; Kusamura S. et al., 2021 [67]; Alyami M. et al., 2021 [73]
Current role	It is the standard of care for peritoneal carcinomatosis, particularly for tumors such as mesothelioma, pseudomyxoma peritonei, and carcinomatosis from appendiceal tumors, endorsed by PSOGI and the Chicago Consensus Group.	Emerging and complementary approach; used for locoregional control or as a “downstaging” strategy before surgery.	PSOGI Consensus; Hübner M. et al., 2024 [70]; Alyami M. et al., 2021 [73]
Future perspectives	Optimization of perfusion protocols; integration with molecular profiling and organoid-guided HIPEC models.	Exploration of combinations with immunotherapy; use in neoadjuvant or maintenance settings; integration with preclinical 3D models.	Löke D.R. et al., 2023 [75]; Alyami M. et al., 2021 [73]; Varinelli L. et al., 2024 [76]

## Data Availability

No new data were created or analyzed in this study.
